# Reporting quality and adherence of randomized controlled trials about statins and/or fibrates for diabetic retinopathy to the CONSORT checklist

**DOI:** 10.1186/s13063-019-3868-4

**Published:** 2019-12-16

**Authors:** Vânia Mozetic, Letícia Leonel, Rafael Leite Pacheco, Carolina de Oliveira Cruz Latorraca, Taís Guimarães, Patricia Logullo, Rachel Riera

**Affiliations:** 1 Ophthalmologist of Instituto Dante Pazzanese de Cardiologia, Sao Paulo, Brazil; 2 Discipline of Evidence-Based Medicine, Universidade Federal de São Paulo (Unifesp), São Paulo, Brasil; 3 Centro Universitário São Camilo, São Paulo, Brazil; 4 EQUATOR Network, NDORMS, Oxford University, Oxford, UK; 5 Centre of Health Technology Assessment, Hospital Sírio-Libanês, São Paulo, Brazil

**Keywords:** Clinical trials as topic, Publication bias, Hydroxymethylglutaryl-CoA reductase inhibitors, Hypolipidemic agents, Diabetic retinopathy

## Abstract

**Background:**

A considerable amount of randomized controlled trials (RCTs) have been published on statins and/or fibrates for diabetic retinopathy, a clinical condition associated with high social and economic burden. Adherence to the CONSORT statement items is imperative to ensure transparency and reproducibility in clinical research. The aim of this study is to assess the reporting quality and the adherence to CONSORT of RCTs assessing statins and/or fibrates for diabetic retinopathy.

**Methods:**

We conducted a critical appraisal study at Discipline of Evidence-based Medicine, Escola Paulista de Medicina, Universidade Federal de São Paulo (Unifesp). A sensitive literature search was performed to identify all relevant RCTs, with no time or language limits. Two authors independently evaluated the reporting quality of the selected RCTs using the CONSORT statement as a standard.

**Results:**

Thirteen reports of RCTs were included in this study. The adherence of the reports to CONSORT items ranged from 24% to 68%. The median score was 11 (interquartile range (IQR) 8 to 13). When analyzed separately, the methods sections of the reports had a median of three items (IQR 2 to 4) judged adherent to the methods items of CONSORT (items 3 to 12). The most underreported items were those related to trial design, title and abstract, allocation concealment, implementation of the randomization sequence, and blinding. Other important items, such as the one related to the description of the inclusion criteria, also had low adherence.

**Conclusions:**

The overall adherence to the CONSORT checklist items was poor, especially in the items related to the methods section. RCT reports on statins and/or fibrates for diabetic retinopathy must be optimized to avoid reporting biases and to improve transparency and reproducibility.

## Background

When assessing the effects of an intervention for a specific clinical condition, randomized controlled trials (RCTs) are considered the preferable source of evidence to support its use [1]. Well-designed, well-conducted, and well-reported RCTs provide the most unbiased data for reducing the uncertainties around effects of an outcome of interest and for improving the reliability of findings [1].

A considerable amount of RCTs have been published on statins and/or fibrates for diabetic retinopathy, a clinical condition associated with high social and economic burden [2]. To ensure their transparency and reproducibility, the International Committee of Medical Journal Editors (ICMJE) recommends the use of reporting guidelines that aim to improve the quality of the reports from studies on healthcare [3, 4].

The CONSORT (CONsolidated Standards of Reporting Trials) [4] statement is used worldwide as a reporting guideline focused on RCTs. The statement was published in 1996 [5], updated in 2010, and consists of a checklist of 25 items that guide the reporting of essential items of a RCT [4]. The CONSORT checklist is divided into six sections: title and abstract (one item), introduction (one item), methods (ten items), results (seven items), discussion (three items), and other information (three items).

Assessing published trials for their completeness—i.e., the adherence to CONSORT checklists—is important for directing further publication policies and for minimizing the risk of selective and/or publication bias [6]. To our knowledge, there has been no such quality evaluation of RCTs on statins and/or fibrates for diabetic retinopathy.

The objective of this study was to evaluate the quality of reporting of RCTs about statin and/or fibrates for diabetic retinopathy by assessing its adherence to the CONSORT checklist.

## Methods

### Design and setting

This is a critical appraisal study performed in the Discipline of Evidence-based Medicine of Escola Paulista de Medicina, Universidade Federal de São Paulo (EPM-Unifesp).

### Criteria for including studies

#### Types of participants

We searched for studies including type 1 or 2 diabetic patients, regardless of age and sex. Participants with or without diabetic retinopathy were considered, depending on the therapeutic or preventive purpose of the related RCT. We considered the diagnosis of diabetic retinopathy by any criteria previously validated.

#### Types of studies

We included RCTs with parallel, cross-over or cluster designs. We only included complete and published studies. Protocols were not included.

#### Types of interventions

We considered statin and/or fibrate, at any dosage, scheme, duration, and route of delivery. As comparators we considered other interventions, no intervention, or placebo.

### Search for studies

We performed a sensitive search strategy without language, date, or publication status restrictions, using relevant descriptors and indexed terms in all databases. An electronic search was performed on January 17, 2018 in the following databases: Medical Literature Analysis and Retrieval System (MEDLINE, via PubMed), Embase (via Elsevier), Latin American and Caribbean Center on Health Sciences Information (LILACS, via Virtual Health Library), and Cochrane Central Register of Controlled Trials (CENTRAL, via Wiley). The full search strategies for each database are presented in Additional file 1**.**

We conducted additional searches in the clinical trial registries ClinicalTrials.gov and International Clinical Trials Registry Platform—World Health Organization (ICTRP-WHO), and in the grey literature source OpenGrey. We also performed a manual search from reference lists of all included studies and review articles for additional studies. We contacted field specialists about unpublished or ongoing studies that could fulfill our inclusion criteria.

### Study selection

The selection of studies was performed by two authors (VM and RP) independently. The first step of the selection was the reading of titles and abstracts. All potentially relevant studies were taken to a second step that consisted of a full text reading. All studies that fulfilled our inclusion criteria were included for critical appraisal. A third author (RR) was consulted if disagreement occurred in any step of the selection process. Selection was performed using Rayyan software [7].

### Data extraction and results presentation

Two authors (VM, RR, or LL) independently extracted data on results of the included studies and transfered data to an a priori developed sheet.

### Assessment of reporting quality

Two authors (TG, LL, or VM) independently assessed the quality of the included studies. A third author (RR or RLP) was consulted in case of disagreement.

For assessing the reporting quality, we used the CONsolidated Standards Of Reporting Trials (CONSORT) statement, a validated tool comprising a checklist of 25 items [8]. We confirmed the adherence to the 25 items and scored each item as: 0 (no adherence) or 1 (full adherence). The final CONSORT-based score achieved by each RCT was determined as a percentage of the maximum possible score. After the exclusion of Not applicable items, if there were any, we present the CONSORT-based score in a 0 to 1 scale.

## Results

### Search results

**Fig. 1:**
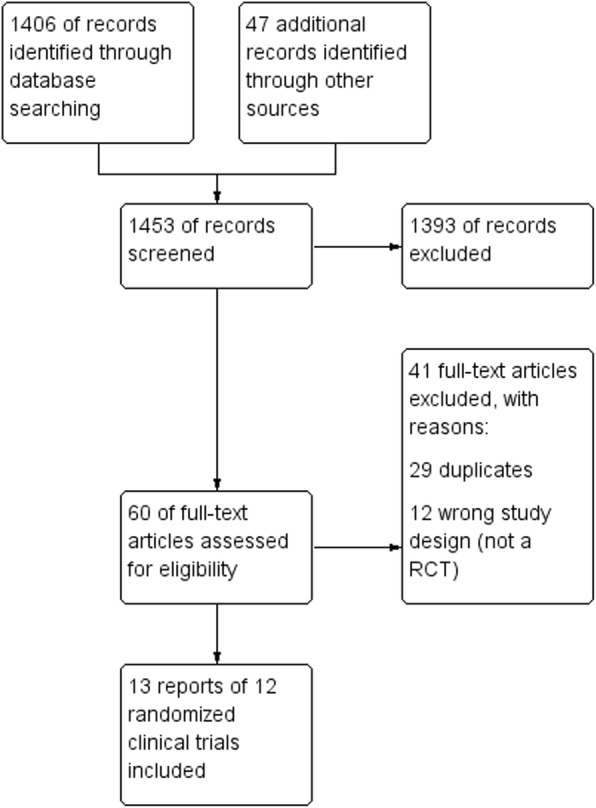
Flow diagram of the selection process

Search strategies retrieved 1408 references. The Preferred Reporting Items for Systematic Reviews and Meta-Analyses (PRISMA) diagram is depicted in Fig. 1**.** After the selection process, 13 reports of RCTs fulfilled the eligibility criteria and were included [9–21].

### Reporting quality of RCTs

**Fig. 2:**
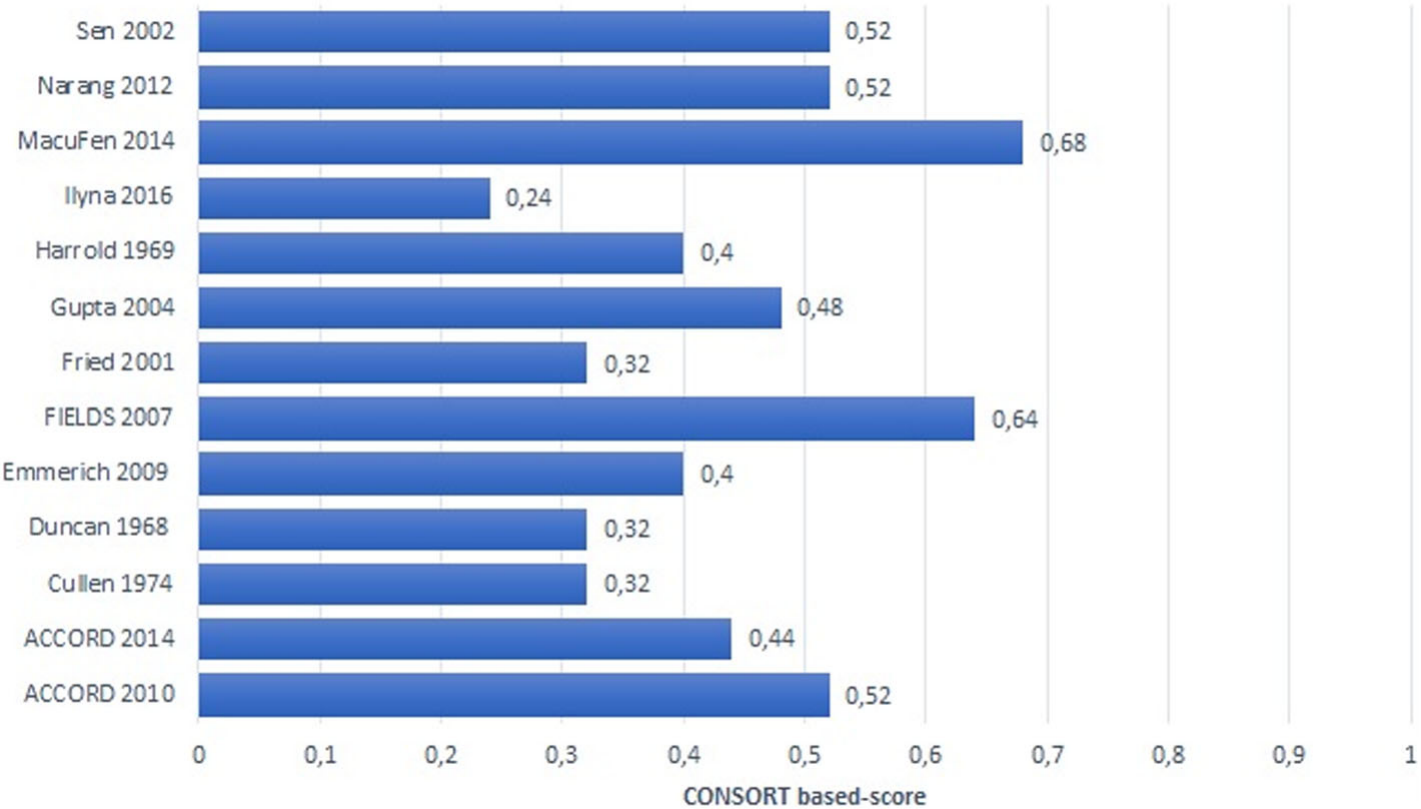
CONSORT-based score for each included randomized controlled trial

The adherence of RCTs to CONSORT items ranged from 24% to 68%. The median score was 11 (interquartile range (IQR) 8 to 13). When analyzed separately, the methods section items of CONSORT (items 3 to 12) had a median of three items judged adequate (IQR 2 to 4). The results section items (items 13 to 19) had a median of four items judged adequate (IQR 4 to 5) Fig. 2 presents the final score for each RCT report. Table 1 presents the number of times that each item was judged adequate. Additional file 2 presents the reporting quality of the included RCTs based on the 25 CONSORT items.

**Table 1:** Number of times each CONSORT item was judged adequate among the 13 included randomized controlled trials

CONSORT item	Number of times judged as adequate (percentage)	95% Confidence interval
1	1 (8%)	(0% to 35%)
2	9 (69%)	(42% to 88%)
3	0 (0%)	(0% to 27%)
4	2 (15%)	(3% to 43%)
5	9 (69%)	(42% to 88%)
6	5 (38%)	(18% to 65%)
7	2 (15%)	(3% to 43%)
8	6 (46%)	(23% to 71%)
9	1 (8%)	(0% to 35%)
10	1 (8%)	(0% to 35%)
11	1 (8%)	(0% to 35%)
12	9 (69%)	(42% to 88%)
13	2 (15%)	(3% to 43%)
14	2 (15%)	(3% to 43%)
15	9 (69%)	(42% to 88%)
16	11 (84%)	(57% to 97%)
17	11 (84%)	(57% to 97%)
18	12 (92%)	(65% to 100%)
19	8 (62%)	(35% to 82%)
20	5 (38%)	(18% to 65%)
21	11 (84%)	(57% to 97%)
22	12 (92%)	(65% to 100%)
23	4 (31%)	(12% to 58%)
24	3 (23%)	(8% to 51%)
25	9 (69%)	(42% to 88%)

Item 3 (“trial design”) was judged adequately reported in none of the RCTs, mainly because they did not specify the allocation ratio. Items 1 (“title and abstract”), 9 (“allocation concealment”), 10 (“implementation” of the randomization sequence), and 11 (“blinding”) were reported adequately in only one RCT. Other important items such as 4 (“participants”), which refers to eligibility criteria and information regarding settings and locations from the RCTs, also had a very low adherence.

## Discussion

This study included 13 reports of randomized clinical trials that assessed statins and/or fibrates for diabetic retinopathy. The adherence to the CONSORT items was poor, and thus the overall reporting quality was judged poor.

Reporting standards for RCTs have been broadly discussed since the first publication of the CONSORT statement and its proposed checklist with 25 items [22]. Inadequate reporting by a scientific study, especially involving experimentation in humans, is scientific misconduct and is associated with resource waste. Adequate reporting means transparency and improves reproducibility [23, 24].

The most underreported items in our study are related to the methods section. Overall, the methods sections were poorly reported and the results and discussion sections were better reported. The RCT with the highest score based on CONSORT adhered to 17 items (68% adherence).

Our findings are similar to other studies that have assessed the overall reporting quality and CONSORT statement checklist adherence in RCTs in a variety of fields [6, 25, 26]. As in other areas of health research, the reproducibility of clinical studies on diabetic retinopathy seems to be impaired by poor reporting. The low quality of reporting affects the synthesis of evidence in systematic reviews as well.

Our study has some limitations. The CONSORT checklist was proposed as a reporting guideline tool for writing, rather than a tool for the evaluation of already published reports. However, since there is no reliable validated tool to assess the reporting quality of RCTs, we believe that a score based on CONSORT is the most reliable option so far. We also planned to assess cluster trials using the original CONSORT items, but there is a CONSORT extension to cluster trials [27]. Nevertheless, the effect of this limitation on our results was probably null since no cluster trials were retrieved by the search strategy. Additionally, most of the included reports (8 out of 13) were published before the last CONSORT update (2010). However, this does not mean that previous RCTs should not adhere to reporting recommendations, because the checklist was already available in 1996 and reviewed in 2001. We did not expect that RCTs published before the updated CONSORT would adhere completely to CONSORT, but we expected that they would report everything that is important for study reproducibility and clinical practice application. Other studies and systematic reviews [6] have looked at the comparison between RCTs published before and after each version of CONSORT, which although important to confirm if the checklist indeed increased the reporting quality of the published studies, was not the objective of our study.

The results from our study should guide future research into diabetic retinopathy. The reporting of future studies must be optimized and their adherence to the CONSORT items is imperative to avoid reporting biases.

## Conclusions

This study included 13 reports of RCTs that assessed statins and/or fibrates for diabetic retinopathy. The overall adherence to the CONSORT checklist items was poor, especially the items related to the methods section. Reporting from future studies must be optimized to avoid reporting biases and to improve transparency and reproducibility.

## Supplementary information

**Additional file 1:** Search strategies for each electronic database.

**Additional file 2:** Adherence to the CONSORT items.

## Data Availability

Data sharing not applicable to this article as no datasets were generated or analyzed during the current study.
