# Supplementary material for: Reporting quality and adherence of randomized controlled trials about statins and/or fibrates for diabetic retinopathy to the CONSORT checklist
Source: Trials. 2019 Dec 16;20:729. doi: 10.1186/s13063-019-3868-4 (PMC6916100; doi:10.1186/s13063-019-3868-4)
Supplement: Supplementary file 1 — Additional file 1: Search strategies for each electronic database. [file 13063_2019_3868_MOESM1_ESM.docx]

**Additional file 1.** Search strategies for each electronic database

| MEDLINE (via Pubmed) | "Hypolipidemic Agents"[Mesh] OR (Hypolipidemic Agents) OR (Agents, Hypolipidemic) OR (Antihyperlipidemics) OR (Hypolipidemic Drugs) OR (Drugs, Hypolipidemic) OR (Antilipemic Drugs) OR (Drugs, Antilipemic) OR (Antilipemics) OR (Antihyperlipemics) OR (Antilipemic Agents) OR (Agents, Antilipemic) OR  "Fibric Acids"[Mesh] OR (Fibric Acids) OR (Methyl-2-Phenoxypropanoic Acid Derivatives) OR (Methyl 2 Phenoxypropanoic Acid Derivatives) OR (Fibric Acid Derivative) OR (Acid Derivatives, Fibric) OR (2-Phenoxy Isobutyric Acids) OR (2 Phenoxy Isobutyric Acids) OR (Isobutyric Acids, 2-Phenoxy) OR (2-Phenoxy-2-Methylpropionic Acid Derivatives) OR (2 Phenoxy 2 Methylpropionic Acid Derivatives) OR (Fibrates) OR  "Fenofibrate"[Mesh] OR (Fenofibrate) OR (Procetofen) OR (Procetofene) OR (Phenofibrate) OR (Apo-Feno-Micro) OR (Apo Feno Micro) OR (Apotex Brand of Procetofen) OR (Apo-Fenofibrate) OR (Apo Fenofibrate) OR (CiL) OR (Lichtenstein Brand of Procetofen) OR (Controlip) OR (Knoll Brand of Procetofen) OR (durafenat) OR (Merck dura Brand of Procetofen) OR (Fenobeta) OR (betapharm Brand of Procetofen) OR (Fenofanton) OR (Q-Pharm Brand of Procetofen) OR (Q Pharm Brand of Procetofen) OR (Anto Brand of Procetofen) OR (Fenofibrat AbZ) OR (AbZ Brand of Procetofen) OR (Fenofibrat AL) OR (Aliud Brand of Procetofen) OR (Fenofibrat AZU) OR (AZU, Fenofibrat) OR (Azupharma Brand of Procetofen) OR (Fenofibrat Heumann) OR (Heumann, Fenofibrat) OR (Heumann Brand of Procetofen) OR (Fenofibrat Hexal) OR (Hexal, Fenofibrat) OR (Hexal Brand of Procetofen) OR (Fenofibrat Stada) OR (Stada, Fenofibrat) OR (Stadapharm Brand of Procetofen) OR (fenofibrat von ct) OR (ct-Arzneimittel Brand of Procetofen) OR (ct Arzneimittel Brand of Procetofen) OR (Fenofibrat-ratiopharm) OR (Fenofibrat ratiopharm) OR (ratiopharm Brand of Procetofen) OR (FÈnofibrate MSD) OR (GNR-Pharma Brand of Procetofen) OR (GNR Pharma Brand of Procetofen) OR (Gen-Fenofibrate) OR (Gen Fenofibrate) OR (Genpharm Brand of Procetofen) OR (LF-178) OR (LF 178) OR (LF178) OR (Lipanthyl) OR (Fenofibrat FPh) OR (Fournier Brand of Procetofen) OR (FÈnofibrate Debat) OR (Debat, FÈnofibrate) OR (Supralip) OR (Lipidil) OR (Lipidil-Ter) OR (Lipidil Ter) OR (Schering-Plough Brand of Procetofen) OR (Schering Plough Brand of Procetofen) OR (Secalip) OR (United Drug Brand of Procetofen) OR (Lipantil) OR (Liparison) OR (Novartis Brand of Procetofen) OR (Livesan) OR (Bouchara Brand of Procetofen) OR (Lofibra) OR (Gate Brand of Procetofen) OR (MTW-Fenofibrat) OR (MTW Fenofibrat) OR (MTW Brand of Procetofen) OR (Novo-Fenofibrate) OR (Novo Fenofibrate) OR (Novopharm Brand of Procetofen) OR (Nu-Fenofibrate) OR (Nu Fenofibrate) OR (Nu-Pharm Brand of Procetofen) OR (Nu Pharm Brand of Procetofen) OR (PMS-Fenofibrate Micro) OR (PMS Fenofibrate Micro) OR (Pharmascience Brand of Procetofen) OR (Tricor) OR (Normalip) OR (Abbott Brand of Procetofen) OR (Antara Micronized Procetofen) OR (Micronized Procetofen, Antara) OR (Procetofen, Antara Micronized) OR (Reliant Brand of Procetofen) OR (Procetofen Reliant Brand) OR (Lipanon) OR  "Bezafibrate"[Mesh] OR (Bezafibrate) OR (Befibrat) OR (Hennig Brand of Bezafibrate) OR (Bezafibrate Hennig Brand) OR (Beza-Lande) OR (Beza Lande) OR (BezaLande) OR (Synthelabo Brand of Bezafibrate) OR (Bezafibrate Synthelabo Brand) OR (Beza-Puren) OR (Beza Puren) OR (BezaPuren) OR (Isis Brand of Bezafibrate) OR (Bezafibrate Isis Brand) OR (Bezabeta) OR (Betapharm Brand of Bezafibrate) OR (Bezafibrate Betapharm Brand) OR (Bezacur) OR (Hexal Brand of Bezafibrate) OR (Bezafibrate Hexal Brand) OR (Bezafibrat PB) OR (PB, Bezafibrat) OR (Teva Brand of Bezafibrate) OR (Bezafibrate Teva Brand) OR (Bezafisal) OR (Cryopharma Brand of Bezafibrate) OR (Bezafibrate Cryopharma Brand) OR (Bezalip) OR (Eulitop) OR (Lakeside Brand of Bezafibrate) OR (Bezafibrate Lakeside Brand) OR (Boehringer Mannheim Brand of Bezafibrate) OR (Bezamerck) OR (durabezur) OR (BM-15.075) OR (BM 15.075) OR (BM15.075) OR (Cedur) OR (Difaterol) OR (Roche Brand of Bezafibrate) OR (Bezafibrate Roche Brand) OR (BÈfizal) OR (Lipox) OR (TAD Brand of Bezafibrate) OR (Bezafibrate TAD Brand) OR (Reducterol) OR (Elfar Brand of Bezafibrate) OR (Bezafibrate Elfar Brand) OR (Regadrin B) OR (Berlin-Chemie Brand of Bezafibrate) OR (Berlin Chemie Brand of Bezafibrate) OR (Bezafibrate Berlin-Chemie Brand) OR (Sklerofibrat) OR (Merckle Brand of Bezafibrate) OR (Bezafibrate Merckle Brand) OR (Solibay) OR (Bayer Brand of Bezafibrate) OR (Bezafibrate Bayer Brand) OR (Azufibrat) OR (Azupharma Brand of Bezafibrate) OR (Bezafibrate Azupharma Brand) OR  "Clofibrate"[Mesh] OR (Clofibrate) OR (Ethyl Chlorophenoxyisobutyrate) OR (Chlorophenoxyisobutyrate, Ethyl) OR (Clofibric Acid, Ethyl Ester) OR (Atromid) OR (Atromid S) OR (Miscleron) OR (Miskleron) OR (Athromidin) OR  "ciprofibrate" [Supplementary Concept] OR (ciprofibrate) OR (Oroxadin) OR (Win 35,833) OR (Lipanor) OR (Modalim) OR (Hyperlipen) OR "Clofenapate"[Mesh] OR (Clofenapate) OR (Methylclofenapate) OR (Methyl Clofenapate) OR (Clofenapate, Methyl) OR "ICI-55695" OR "ICI 55695" OR "ICI55695" OR  "Gemfibrozil"[Mesh] OR (Gemfibrozil) OR (Gemfibrosil) OR (Ausgem) OR (Sigma Brand of Gemfibrozil) OR (Bolutol) OR (Farmasierra Brand of Gemfibrozil) OR (Chem mart Gemfibrozil) OR (Chem mart Brand of Gemfibrozil) OR (CI-719) OR (CI 719) OR (CI719) OR (DBL Gemfibrozil) OR (Bull Brand of Gemfibrozil) OR (Decrelip) OR (Ferrer Brand of Gemfibrozil) OR (Gemfi 1A Pharma) OR (1A Brand of Gemfibrozil) OR (Gemfibrozilo Bayvit) OR (Bayvit, Gemfibrozilo) OR (Bayvit Brand of Gemfibrozil) OR (Gemfibrozilo Bexal) OR (Bexal Brand of Gemfibrozil) OR (Gemfibrozilo Ur) OR (Cantabria Brand of Gemfibrozil) OR (Gemhexal) OR (Hexal Brand of Gemfibrozil) OR (Gen-Gemfibrozil) OR (Gen Gemfibrozil) OR (GenGemfibrozil) OR (Genpharm Brand of Gemfibrozil) OR (GenRX Gemfibrozil) OR (Gemfibrozil, GenRX) OR (Faulding Brand of Gemfibrozil) OR (Healthsense Gemfibrozil) OR (Gemfibrozil, Healthsense) OR (Healthsense Brand of Gemfibrozil) OR (Jezil) OR (Alphapharm Brand of Gemfibrozil) OR (Lipazil) OR (Douglas Brand of Gemfibrozil) OR (Lipox Gemf) OR (TAD Brand of Gemfibrozil) OR (Litarek) OR (Ipsen Brand of Gemfibrozil) OR (Lopid) OR (Lopid R) OR (Warner-Lambert Brand of Gemfibrozil) OR (Warner Lambert Brand of Gemfibrozil) OR (Pfizer Brand of Gemfibrozil) OR (United Drug Brand of Gemfibrozil) OR (Lipur) OR (Parke Davis Brand of Gemfibrozil) OR (Novo-Gemfibrozil) OR (Novo Gemfibrozil) OR (Novopharm Brand of Gemfibrozil) OR (Nu-Gemfibrozil) OR (Nu Gemfibrozil) OR (NuGemfibrozil) OR (Nu-Pharm Brand of Gemfibrozil) OR (Nu Pharm Brand of Gemfibrozil) OR (Pilder) OR (Quimifar Brand of Gemfibrozil) OR (PMS-Gemfibrozil) OR (PMS Gemfibrozil) OR (Pharmascience Brand of Gemfibrozil) OR (SBPA Gemfibrozil) OR (Gemfibrozil, SBPA) OR (Biochemie Brand of Gemfibrozil) OR (Terry White Chemists Gemfibrozil) OR (Terry White Chemists Brand of Gemfibrozil) OR (Trialmin) OR (Menarini Brand of Gemfibrozil) OR (Apo-Gemfibrozil) OR (Apo Gemfibrozil) OR (ApoGemfibrozil) OR (Apotex Brand of Gemfibrozil) OR  "simfibrate" [Supplementary Concept] OR (simfibrate) OR "1,3-propanediol bis(alpha-p-chlorophenoxyisobutyrate)" OR (CLY 503) OR (CLY-503) OR (Cholesolvin) OR "etofibrate" [Supplementary Concept] OR (etofibrate) OR (Lipo-Merz) OR (Tricerol) OR (etofibrate hydrochloride) OR  "clofibride" [Supplementary Concept] OR (clofibride) OR "N-dimethylbutyramide 4-chlorophenoxyisobutyrate" OR "4-hydroxy-N,N-dimethylbutyramide 4-chlorophenoxyisobutyrate" OR "MG 46" OR (lipenan) OR  "pirifibrate" [Supplementary Concept] OR (pirifibrate) OR (Bratenol) OR "EL-466" OR "(6-(hydroxymethyl)-2-pyridyl)methyl-2-(4-chlorophenoxy)-2-methyl-propionate hydrochloride" OR "dulofibrate" [Supplementary Concept] OR (dulofibrate) OR "4-chlorophenyl 2-(4-chlorophenoxy)isobutyrate" OR "2-(4-chlorophenoxy)isobutyric acid 4-chlorophenyl ester" OR  "clinofibrate" [Supplementary Concept] OR (clinofibrate) OR "1,1-bis(4'-(1''-carboxy-1''-methylpropoxy)phenyl)cyclohexane" OR "S 8527" OR "clinofibrate, (R*,R*)-(-)-isomer" OR "clinofibrate, (R*,S*)-isomer" OR "clinofibrate, (R*,R*)-(+)-isomer" OR "clinofibrate, (R*,R*)-(+-)-isomer" OR  "lifibrate" [Supplementary Concept] OR (lifibrate) OR "1-methyl-4-piperidyl bis(4-chlorophenoxy)acetate" OR "SaH 42-348" OR "picafibrate" [Supplementary Concept] OR (picafibrate) OR "(4-chloro-2-phenoxy)-2-methyl-2-propionyl-2- ethylnicotinamide" OR "F 1393" OR  ronifibrate OR biclofibrate OR fenirofibrate OR nicofibrate OR ponfibrate OR salafibrate OR serfibrate OR tiafibrate OR timofibrate OR "tocofibrate" [Supplementary Concept] OR (tocofibrate) OR "tocopheryl 2-(p-chlorophenoxy)isobutyrate" OR  "Hydroxymethylglutaryl-CoA Reductase Inhibitors"[Mesh] OR (Hydroxymethylglutaryl-CoA Reductase Inhibitors) OR (Hydroxymethylglutaryl CoA Reductase Inhibitors) OR (Inhibitors, Hydroxymethylglutaryl-CoA Reductase) OR (Reductase Inhibitors, Hydroxymethylglutaryl-CoA) OR (Inhibitors, HMG-CoA Reductase) OR (Inhibitors, HMG CoA Reductase) OR (Inhibitors, Hydroxymethylglutaryl-Coenzyme A) OR (Hydroxymethylglutaryl-Coenzyme A Inhibitors) OR (Inhibitors, Hydroxymethylglutaryl Coenzyme A) OR (Statins, HMG-CoA) OR (HMG-CoA Statins) OR (Statins, HMG CoA) OR (HMG-CoA Reductase Inhibitors) OR (HMG CoA Reductase Inhibitors) OR (Reductase Inhibitors, HMG-CoA) OR (Inhibitors, Hydroxymethylglutaryl-CoA) OR(Hydroxymethylglutaryl-CoA Inhibitors) OR (Inhibitors, Hydroxymethylglutaryl CoA) OR  "Simvastatin"[Mesh] OR (Simvastatin) OR (Synvinolin) OR (MK-733) OR (MK 733) OR (MK733) OR (Zocor) OR (SINVALIP) OR (VASLIP) OR "ezetimibe, simvastatin drug combination" [Supplementary Concept] OR (ezetimibe, simvastatin drug combination) OR (ezetimibe-simvastatin combination) OR (Vytorin) OR (Inegy) OR  "Lovastatin"[Mesh]] OR (Lovastatin) OR (Mevinolin) OR (Monacolin K) OR (6-Methylcompactin) OR (6 Methylcompactin) OR (MK-803) OR (MK 803) OR (MK803) OR (Mevacor) OR "Lovastatin, (1 alpha(S*))-Isomer" OR (Lovastatin, 1 alpha-Isomer) OR (1 alpha-Isomer Lovastatin) OR (Lovastatin, 1 alpha Isomer) OR (alpha-Isomer Lovastatin, 1) OR  "Pravastatin"[Mesh] OR (Pravastatin) OR (Eptastatin) OR (Vasten) OR (Aventis Brand of Pravastatin Sodium) OR (CS-514) OR (CS 514) OR (CS514) OR (Lin-Pravastatin) OR (Lin Pravastatin) OR (Linson Pharma Brand of Pravastatin Sodium) OR (Lipemo) OR (Squibb Brand of Pravastatin Sodium) OR (Liplat) OR (Esteve Brand of Pravastatin Sodium) OR (Nu-Pravastatin) OR (Nu Pravastatin) OR (Nu-Pharma Brand of Pravastatin Sodium) OR (Prareduct) OR (Sankyo Brand of Pravastatin Sodium) OR (Mevalotin) OR (Pravachol) OR (Elisor) OR (Selektine) OR (Pravacol) OR (Pravasin) OR (Bristol-Myers Squibb Brand of Pravastatin Sodium) OR (Lipostat) OR (Pravastatin Monosodium Salt, (6 beta)-Isomer) OR (Pravastatin Sodium) OR (Pravastatin Sodium Salt) OR (Sodium Salt, Pravastatin) OR (Pravastatin tert-Octylamine Salt) OR (Pravastatin tert Octylamine Salt) OR (Pravastatin, (6 beta)-Isomer) OR (RMS-431) OR (RMS 431) OR (RMS431) OR (SQ-31000) OR (SQ 31000) OR (SQ31000) OR (SQ-31,000) OR (SQ 31,000) OR (SQ31,000) OR (Apo-Pravastatin) OR (Apo Pravastatin) OR (Apotex Brand of Pravastatin Sodium) OR (Bristacol) OR (Juste Brand of Pravastatin Sodium) OR  "fluvastatin" [Supplementary Concept] OR (fluvastatin) OR (fluindostatin) OR "7-(3-(4-fluorophenyl)-1-(1-methylethyl)-1H-indol-2-yl)-3,5-dihydroxy-6-heptenoate" OR (Lescol) OR (XU 62-320) OR (XU-62320) OR (XU 62320) OR (fluvastatin sodium) OR (fluvastatin sodium salt) OR  "atorvastatin" [Supplementary Concept] OR (atorvastatin) OR (liptonorm) OR (Lipitor) OR (CI 981) OR (CI-981) OR (atorvastatin calcium) OR (atorvastatin calcium hydrate) OR (atorvastatin calcium trihydrate) OR (atorvastatin, calcium salt) OR (atorvastatin calcium anhydrous) OR  "cerivastatin" [Supplementary Concept] OR (cerivastatin) OR (Kazak) OR (cerivastatin sodium) OR "6-Heptenoic acid, 7-(4-(4-fluorophenyl)-5-(methoxymethyl)-2,6-bis(1-methylethyl)-3-pyridinyl)-3,5-dihydroxy-, monosodium salt, (S-(R*,S*-(E)))-" OR "7-(4-(4-fluorophenyl)-2,6-diisopropyl-5-(methoxymethyl)pyrid-3-yl)-3,5-dihydroxy-6-heptenoate sodium salt" OR (rivastatin) OR (Certa) OR (Bay w 6228) OR (Baycol) OR (Lipobay) OR  "rosuvastatin" [Supplementary Concept] OR (rosuvastatin) OR (rosuvastatin calcium) OR (ZD4522) OR (ZD 4522) OR (Crestor) OR "pitavastatin" [Supplementary Concept] OR (pitavastatin) OR (nisvastatin) OR (itavastatin) OR (P 872441) OR (P-872441) OR (NK 104) OR (NK-104) OR  "mevastatin" [Supplementary Concept] OR (mevastatin) OR (compactin) OR "6-demethylmevinolin" OR "CS 500" OR "ML 236B" OR "ML236B" OR "ML-236B" OR "crilvastatin" [Supplementary Concept] OR (crilvastatin) OR  "dalvastatin" [Supplementary Concept] OR (dalvastatin) OR "6-(2-(2-(4-fluoro-3-methylphenyl)-4,4,6,6-tetramethyl-1-cyclohexen-1-yl)ethenyl)tetrahydro-4-hydroxy-2H-pyran-2-one" OR "RG 12561" OR "RG-12561" OR  "mevinolinic acid" [Supplementary Concept] OR (mevinolinic acid) OR (lovestatin acid) OR "mevinolinic acid, (1S-(1alpha(betaS*,deltaS*),2alpha,6beta,8beta(R*),8aalpha))-isomer" OR "mevinolinic acid, aluminum salt (3:1)" OR "mevinolinic acid, monoammonium salt, 1S-(1alpha(betaS*),2alpha,6beta,8beta(R*),8aalpha)-isomer" OR (mevinolinic acid, monosodium salt) OR "mevinolinic acid, monosodium salt, (1S-(1alpha(betaS*,deltaS*),2alpha,6beta,8beta(R*),8aalpha))-isomer" OR "L 154819" OR "L-154819" OR "L-154,819" OR "mevinolinic acid, calcium salt (2:1)" OR  "monacolin J" [Supplementary Concept] OR (monacolin J) OR "monacolin L" [Supplementary Concept] OR (monacolin L) OR glenvastatin OR bervastatin  AND  "Diabetic Retinopathy"[Mesh] OR (Diabetic Retinopathy) OR (Diabetic Retinopathies) OR (Retinopathies, Diabetic) OR (Retinopathy, Diabetic)  #28 #26 AND #27 |
| --- | --- |
| Embase (via Elsevier) | #1 'antilipemic agent'/exp OR (anti hyperlipidemic agent) OR (anti hyperlipidemic drug) OR (antihyperlipidemic agent) OR (antihyperlipidemic drug) OR (antihyperlipidemics) OR (antilipaemia agent) OR (antilipaemia drug) OR (antilipaemic agent) OR (antilipaemic agents) OR (antilipaemic drug) OR (antilipemia agent) OR (antilipemia drug) OR (antilipemic agents) OR (antilipemic drug) OR (antilipidemic agent) OR (antilipidemic drug) OR (hypolipaemic agent) OR (hypolipemic agent) OR (hypolipidemic agent) OR (hypolipidemic agents) OR (hypolipidemic drug) OR (lipid depressing agent) OR (lipid depressing drug) OR (lipid lowering agent) OR (lipid lowering drug)  #2 'fibric acid derivative'/exp OR (fibrate) OR (fibrate derivative) OR (fibrates) OR (fibric acid) OR (fibric acid derivatives) OR (fibric acids)  #3 'fenofibrate'/exp OR "2 [4 (4 chlorobenzoyl) phenoxy] 2 methylpropionic acid isopropyl ester" OR (antara) OR "antara (micronized)" OR apo-feno-micro OR (aterolis) OR (bisterol sr) OR (climage) OR (controlip) OR (durafenat) OR (durafenat micro) OR (evothyl) OR (fegenor) OR (felosma) OR (fenobrate) OR (fenofanton) OR "fenofibrate (micronized)" OR (fenogal) OR (fenogal lidose) OR (fenoglide) OR (fenox) OR (fibrafen) OR (grs 001) OR (hyperchol) OR "isopropyl 2 [4 (4 chlorobenzoyl) phenoxy] 2 methylpropionate" OR (katalip) OR (lexemin) OR (lipanthyl) OR (lipanthyl retard) OR (lipantil) OR (lipantyl) OR (liparison) OR (lipidax) OR (lipidil) OR (lipidil supra) OR (lipilo) OR (lipirex) OR (lipoclar) OR (lipofen) OR (lipolin) OR (liposit) OR (lipsin) OR (livesan ge) OR (lofibra) OR (nopid 200) OR (normalip) OR (nubrex) OR (procetofen) OR (procetofenate) OR (procetofene) OR (proketofen) OR (qualipantyl) OR (rapidil) OR (redose 200) OR (rorit) OR (secalip) OR (sigurtil) OR (trichol) OR (tricor) OR "tricor (micronized)" OR (tricor 500) OR (triglide) OR (trolip) OR (zerlubron) OR (zumafib)  #4 'bezafibrate'/exp OR "2 [4 [2 (4 chlorobenzamido) ethyl] phenoxy] 2 methylpropanoic acid" OR "2 [4 [2 (4 chlorobenzamido) ethyl] phenoxy] 2 methylpropionic acid" OR (befizal) OR (benzafibrate) OR (benzofibrate) OR (bezafibrate retard) OR (bezalip) OR (bezalip retard) OR (bezatol) OR (bezifal) OR (bezofibrate) OR (bf 759) OR (bf759) OR (bm 15.075) OR (bm 15075) OR (bm15.075) OR (bm15075) OR (cedur) OR (cedur retard) OR (lo 44) OR (lo44) OR (norlip)  #5 'clofibrate'/exp OR "2 (4 chlorophenoxy) isobutyric acid ethyl ester" OR "2 (para chlorophenoxy) 2 methylpropionic acid ethyl ester" OR "4 chlorophenoxyisobutyric acid ethyl ester" OR (abitrate) OR "alpha (4 chlorophenoxy) isobutyric acid ethyl ester" OR "alpha (para chlorophenoxy) isobutyric acid ethyl ester" OR "alpha (para chlorophenoxy) isobutyrate ethyl ester" OR "alpha (para chlorophenoxy) isobutyric acid ethyl ester" OR "alpha chlorophenoxyisobutyrate ethyl ester" OR "alpha para chlorophenoxyisobutyrate ethyl ester" OR "alpha (para chlorophenoxy) isobutyric acid ethyl ester" OR (amadol) OR (amotril) OR (apolan) OR (apoterin a) OR (arterioflexin) OR (arterol) OR (ateriosan) OR (aterosol) OR (atheropront) OR (atromid s) OR (atromid-s) OR (atromid-s 500) OR (atromidin) OR (atromidine) OR (ay 61123) OR (ay61123) OR (chlorfenisate) OR (chlorphenisate) OR (chlorphenoxyisobutyrate) OR (cholenal) OR (claripex) OR (cpib) OR (clofi icn) OR (clofibrate ethyl ester) OR (clofibric acid ethyl ester) OR (clofinit) OR (clofiral) OR (clofirem) OR (colebron) OR (elpi 500) OR "ethyl 2 (para chlorophenoxy) 2 methylpropionate" OR "ethyl 2 (para chlorophenoxy) isobutyrate" OR "ethyl alpha (para chlorophenoxy) isobutyric acid" OR "ethyl alpha (roh) butyrate" OR "ethyl alpha chlorophenoxyisobutyrate" OR "ethyl alpha para chlorophenoxyisobutyrate" OR "ethyl chlorophenoxyisobutyrate" OR "ethyl p chlorophenoxyisobutyrate" OR "ethyl para chlorophenoxyisobutyrate" OR "ethyl para chlorophenoxyisobutyreate" OR "ethyl para chlorophenoxyisobutyric acid" OR (ici 28257) OR (ici28257) OR (klofiran) OR (lipavlon) OR (lipilim) OR (liprinal) OR (miscleron) OR (misclerone) OR (myscleron) OR (mysclerone) OR (negalip) OR (neo atromid) OR (normolipol) OR (novofibrate) OR (nsc 79389) OR (nsc79389) OR (regelan) OR (regelan n) OR (serotinex) OR (skleromexe) OR (triglicer) OR (yuclo)  #6 'ciprofibrate'/exp OR "2 [4 (2, 2 dichlorocyclopropyl) phenoxy] 2 methylpropanoic acid" OR "2 [4 (2, 2 dichlorocyclopropyl) phenoxy] 2 methylpropionic acid" OR (lipanor) OR (modalim) OR (win 35833)  #7 'methylclofenapate'/exp OR "2 (4 chlorobiphenyl 1 yloxy) 2 methylpropionic acid methyl ester" OR "2 [4 (4 chlorophenyl) phenoxy] 2 methylpropionic acid methyl ester" OR (clofenapate) OR (ici 55695) OR (methyl clofenapate)  #8 'gemfibrozil'/exp OR "2, 2 dimethyl 5 (2, 5 xylyloxy) pentanoic acid" OR "2, 2 dimethyl 5 (2, 5 xylyloxy) valeric acid; 5 (2, 5 dimethylphenoxy) 2, 2 dimethylvaleric acid" OR (apo-gemfibrozil) OR (ausgem) OR (bolutol) OR (brozil) OR (chlorestrol) OR (cholespid) OR (ci 719) OR (ci719) OR (clearol) OR (decrelip) OR (detrichol) OR (elmogan) OR (fetinor) OR (fibralip) OR (fibrocit) OR (gedum) OR (gemfi) OR (gemfibril) OR (gemfibromax) OR (gemizol) OR (gemlipid) OR (gemnpid) OR (gemzil) OR (genfibrozil) OR (gevilon) OR (gevilon uno) OR (gozid) OR (grifogemzilo) OR (hidil) OR (hipolixan) OR (ipolipid) OR (jezil) OR (lanaterom) OR (lifibron) OR (lipazil) OR (lipidys) OR (lipigem) OR (lipira) OR (lipison) OR (lipistorol) OR (lipizyl) OR (lipofor) OR (lipolo) OR (lipostorol) OR (lipozid) OR (lipozil) OR (lipur) OR (lopid) OR (lopid o.d.) OR (lopid tc) OR low-lip OR (lowin) OR (manobrozil) OR (mariston) OR (mersikol) OR (normolip) OR (panazil) OR (polyxit) OR (progemzal) OR (recozil) OR (reducel) OR (regulip) OR (synbrozil) OR (triglizil) OR (uragem) OR (zilop)  #9 'simfibrate'/exp OR (cholesolvin) OR "cholesolvin (tn)" OR (clofibric acid trimethylene diester) OR (cly 503) OR (cly503)  #10 'etofibrate'/exp OR (etolipe) OR (lipo merz) OR (lipo merz retard) OR (lipomerz) OR "nicotinic acid 2 [2 (4 chlorophenoxy) 2 methylpropionoxy] ethyl ester"  #11 'clofibride'/exp OR "clofibric acid 3 (dimethylcarbamoylpropyl) ester" OR (lipenan) OR (mg 46)  #12 'pirifibrate'/exp OR "2, 6 pyridinedimethanol clofibrate" OR "6 (hydroxymethyl) pyrid 2 ylmethyl 2 (4 chlorophenoxy) 2 methylpropionate" OR (bratenol) OR "clofibric acid 2, 6 pyridinedimethanol ester"  #13 'clinofibrate'/exp OR "1, 1 bis [4 (1 carboxy 1 methylpropoxy) phenyl] cyclohexane" OR "2, 2 [cyclohexylidenebis (1, 4 phenyleneoxy)] bis (2 methylbutyric acid)" OR (lipoclin) OR (s 8527)  #14 'dulofibrate'/exp OR "2 (4 chlorophenoxy) 2 methylpropionic acid 4 chlorophenyl ester" OR "4 chlorophenyl 2 (4 chlorophenoxy) 2 methylpropionate" OR "clofibric acid 4 chlorophenyl ester" OR (duclofibrate)  #15 'lifibrate'/exp OR "1 methyl 4 piperidyl bis (4 chlorophenoxy) acetate" OR "1 methyl 4 piperidyl bis (para chlorophenoxy) acetate" OR "1 methyl 4 piperidyl glyoxylate bis (4 chlorophenyl) acetal" OR "1 methylpiperid 4 yl bis (4 chlorophenoxy) acetate" OR "bis (4 chlorophenoxy) acetic acid n methylpiperid 4 yl ester" OR (sah 42-348) OR (sah 42348) OR sah-42-348 OR sah42-348 OR (sah42348)  #16 'picafibrate'/exp OR "2 (4 chlorophenoxy) 2 methylpropionic acid ester with n (2 hydroxyethyl) nicotinamide" OR "2 nicotinamidoethyl clofibrate" OR "clofibric acid 2 nicotinamidoethyl ester"  #17 'ronifibrate'/exp OR "(3 nicotinoyloxypropyl) 2 (4 chlorophenoxy) isobutyrate" OR "(3 nicotinoyloxypropyl) clofibrate" OR "2 (4 chlorophenoxy) 2 methylpropionic acid 3 nicotinoyloxypropyl ester" OR "2 (4 chlorophenoxy) isobutyric acid 3 nicotinoyloxypropyl ester" OR "3 nicotinoyloxypropyl 2 (4 chlorophenoxy) isobutyrate" OR "3 nicotinoyloxypropyl alpha (para chlorophenoxy) isobutyrate" OR "3 nicotinoyloxypropyl clofibrate" OR "clofibric acid 3 nicotinoyloxypropyl ester" OR (cloprane)  #18 'biclofibrate'/exp OR "1 methyl 2 pyrrolidinylmethyl bis (4 chlorophenoxy) acetate" OR "1 methylpyrrolidin 2 ylmethyl bis (4 chlorophenoxy) acetate" OR "2, 2 bis (4 chlorophenoxy) acetic acid (1 methyl 2 pyrrolidinyl) methyl ester" OR "bis (4 chlorophenoxy) acetic acid (1 methyl 2 pyrrolidinyl) methyl ester" OR "bis (4 chlorophenoxy) acetic acid 1 methyl 2 pyrrolidinylmethyl ester"  #19 'fenirofibrate'/exp OR "2 [4 (4 chloro alpha hydroxybenzyl) phenoxy] 2 methylpropanoic acid" OR "2 [4 [(4 chlorophenyl) (hydroxy) methyl] phenoxy] 2 methylpropanoic acid" OR "2 [[alpha (4 chlorophenyl) alpha hydroxy para tolyl] oxy] 2 methylpropionic acid" OR "reduced fenofibric acid"  #20 'nicofibrate'/exp OR "3 hydroxymethylpyridine 4 chlorophenoxyisobutyrate" OR "3 pyridinemethanol ester of alpha (4 chlorophenoxy) isobutyric acid hydrochloride" OR "3 pyridylmethyl 2 (4 chlorophenoxy) 2 methylpropionate" OR (clofenpyrid) OR "clofibric acid 3 pyridylmethyl ester" OR "clofibric acid nicotinyl alcohol ester" OR "clofibric acid pyrid 3 ylmethyl ester"  #21 'ponfibrate'/exp OR "2, 10 dichloro 12 methyldibenzo [d, g] [1, 3] dioxocine 6 carboxylic acid ethyl ester" OR "2, 10 dichloro 12beta methyl 12h dibenzo [d, g] [1, 3] dioxocin 6alpha carboxylic acid ethyl ester" OR "2, 10 dichloro 6 ethoxycarbonyl 12 methyldibenzo [d, g] [1, 3] dioxocin" OR "2, 10 dichloro 6alpha ethoxycarbonyl 12beta methyl 12h dibenzo [d, g] [1, 3] dioxocin" OR (crismel) OR "ethyl 2, 10 dichloro 12 methyldibenzo [d, g] [1, 3] dioxocine 6 carboxylate" OR "trans 2, 10 dichloro 12 methyl 12h dibenzo [d, g] [1, 3] dioxocin 6 carboxylic acid ethyl ester"  #22 'salafibrate'/exp OR "2 (4 chlorophenoxy) 2 methylpropanoic acid 2 [[2 (acetyloxy) benzoyl] oxy] 1, 3 propanediyl ester" OR "2 hydroxy 1 (hydroxymethyl) ethyl salicylate 2 acetate bis [2 (4 chlorophenoxy) 2 methylpropionate]" OR "glycerol 2 (acetylsalicylate) 1, 3 di (clofibrate)" OR "glyceryl 2 (acetylsalicylate) 1, 3 di (clofibrate)"  #23 'serfibrate'/exp OR "n acetyl s [2 (4 chlorophenoxy) 2 methyl 1 oxopropyl] homocysteine" OR "n acetyl s [2 (4 chlorophenoxy) 2 methylpropanoyl] homocysteine" OR "n acetyl s [2 (4 chlorophenoxy) 2 methylpropionyl] homocysteine"  #24 'tiafibrate'/exp OR "2 [10 [2 [2 (4 chlorophenoxy) 2 methylpropanoyl] oxyethylsulfanyl] decylsulfanyl] ethyl 2 (4 chlorophenoxy) 2 methylpropanoate" OR "2, 2 (decamethylenedithio) ethanol bis [2 (4 chlorophenoxy) 2 methylpropionate]" OR "bis [2 (4 chlorophenoxy) 2 methylpropionic acid] 1, 10 decanediylbis (thioethylene) ester"  #25 'timofibrate'/exp OR "3 [2 (4 chlorophenoxy) 2 methyl 1 oxopropyl] 4 thiazolidinecarboxylic acid" OR "3 [2 (4 chlorophenoxy) 2 methylpropionyl] 4 thiazolidinecarboxylic acid"  #26 'tocofibrate'/exp OR "2 (4 chlorophenoxy) 2 methylpropanoic acid 3, 4 dihydro 2, 5, 7, 8 tetramethyl 2 (4, 8, 12 trimethyltridecyl) 2h 1 benzopyran 6 yl ester" OR "2, 5, 7, 8 tetramethyl 2 (4, 8, 12 trimethyltridecyl) 6 chromanyl 2 (4 chlorophenoxy) 2 methylpropionate" OR "3, 4 dihydro 2, 5, 7, 8 tetramethyl 2 (4, 8, 12 trimethyltridecyl) 2h 1 benzopyran 6 yl 2 (4 chlorophenoxy) 2 methylpropionate" OR "alpha tocopherol clofibrate" OR "alpha tocopheryl clofibrate" OR (tocopherol clofibrate) OR (tocopheryl clofibrate) OR (vitamin E clofibrate)  #27 'hydroxymethylglutaryl coenzyme A reductase inhibitor'/exp OR (HMG CoA reductase inhibitor) OR (HMG CoA reductase inhibitors) OR (hmg coenzyme a reductase inhibitor) OR (hmg-coa reductase inhibitors) OR (hydroxymethylglutaryl coa reductase inhibitors) OR (hydroxymethylglutaryl-coa reductase inhibitors) OR "statin (drug)" OR (statins) OR (vastatin)  #28 'simvastatin'/exp OR (avastinee) OR (cholestat) OR (clinfar) OR (colastatina) OR (colestricon) OR (covastin) OR (denan) OR (epistatin) OR (esvat) OR (ethicol) OR (eucor) OR (ifistatin) OR (kavelor) OR (klonastin) OR (kolestevan) OR (l 644128) OR (l644128) OR (lipecor) OR (lipex) OR (lipinorm) OR (liponorm) OR (lipovas) OR (lodales) OR (medipo) OR (mersivas) OR (mk 733) OR (mk733) OR nor-vastina OR (normofat) OR (orovas) OR (rechol) OR (simbado) OR (simcard) OR (simchol) OR (simovil) OR (simtin) OR (simvacor) OR (simvahex) OR (simvalord) OR (simvastar) OR (simvata) OR (simvatin) OR (simvor) OR (simvotin) OR (sinvacor) OR (sinvastatin) OR (sinvinolin) OR (sivastin) OR (starzoco) OR (synvinolin) OR (torio) OR (valemia) OR (vasilip) OR (vasotenal) OR (vazim) OR (vidastat) OR (zimmex) OR (zocor) OR (zocor forte) OR (zocord) OR (zovast)  #29 'mevinolin'/exp OR (altocor) OR (altoprev) OR (artein) OR (belvas) OR (birotin) OR (cholestra) OR (cysin) OR (ellanco) OR (elstatin) OR (l 654969) OR (lipdip) OR (lipivas) OR (lofacol) OR (lomar) OR (lostatin) OR (lovacel) OR (lovacol) OR (lovalip) OR (lovalord) OR (lovastan) OR (lovastatin) OR (lovasterol) OR (lovastin) OR (lovatadin) OR (lowachol) OR (lozutin) OR (medostatin) OR (mevacor) OR (meverstin) OR (mevinacor) OR (mk 0803) OR (mk 803) OR (mk0803) OR (mk803) OR (monacolin K) OR (monakolin k) OR (msd 803) OR (neolipid) OR (nergadan) OR (ovasta) OR (rodatin) OR (rovacor) OR (taucor)  #30 'pravastatin'/exp OR "3, 5 dihydroxy 7 [1, 2, 6, 7, 8, 8a hexahydro 6 hydroxy 2 methyl 8 (2 methylbutyryloxy) 1 naphthyl] heptanoic acid" OR "7 [1, 2, 6, 7, 8, 8a hexahydro 6 hydroxy 2 methyl 8 (2 methylbutyryloxy) 1 naphthyl] 3, 5 dihydroxyheptanoic acid" OR (astin) OR (bristacol) OR (cholespar) OR (cs 514) OR (cs514) OR (elisor) OR (epatostantin) OR (eptastatin) OR (eptastatin sodium) OR (eptastatine) OR (kenstatin) OR (lipemol) OR (lipidal) OR (liplat) OR (lipostat) OR (liprevil) OR (mevalotin) OR (novales) OR (prareduct) OR (prascolend) OR (prastan) OR (prava) OR (pravachol) OR (pravacol) OR (pravaselect) OR (pravasin) OR (pravasine) OR (pravastatin natrium mayrho fer) OR (pravastatin sodium) OR (pravator) OR (pravyl) OR (sanaprav) OR (selektine) OR (selipran) OR (sq 31000) OR (sq31000) OR (stanidine) OR (vasopran) OR (vasten) OR (xipral)  #31 'fluindostatin'/exp OR "7 [3 (4 fluorophenyl) 1 isopropyl 2 indolyl] 3, 5 dihydroxy 6 heptenoic acid" OR (canef) OR (cranoc) OR (fluindostatin sodium) OR (fluvastatin) OR (fluvastatin sodium) OR (fractal lp) OR (lescol) OR (lescol lp) OR (lescol xl) OR (leucol) OR (lochol) OR (locol) OR (sri 62320) OR (sri62320) OR (vastin) OR (xu 62320) OR (xu62320)  #32 'atorvastatin'/exp OR "2 (4 fluorophenyl) beta, delta dihydroxy 5 isopropyl 3 phenyl 4 phenylcarbamoyl 1h pyrrole 1 heptanoic acid" OR (atorlip) OR (atorvastatin calcium) OR (atorvastatin calcium trihydrate) OR (atovarol) OR (cardyl) OR (ci 981) OR (ci981) OR (glustar) OR (lipibec) OR (lipitor) OR (liprimar) OR (liptonorm) OR (lowlipen) OR (sortis) OR (storvas) OR (tahor) OR (torvast) OR (totalip) OR (xarator) OR (ym 548) OR (ym548) OR (zarator)  #33 'cerivastatin'/exp OR "7 [4 (4 fluorophenyl) 2, 6 diisopropyl 5 (methoxymethyl) 3 pyridinyl] 3, 5 dihydroxy 6 heptenoic acid" OR (bay w 6228) OR (bay w6228) OR (baycol) OR (cerivastatin sodium) OR (certa) OR (lipobay) OR (rivastatin)  #34 'rosuvastatin'/exp OR "7 [4 (4 fluorophenyl) 6 isopropyl 2 (n methylmethanesulfonamido) 5 pyrimidinyl] 3, 5 dihydroxy 6 heptenoic acid" OR "7 [4 (4 fluorophenyl) 6 isopropyl 2 (n methylmethanesulfonamido) pyrimidin 5 yl] 3, 5 dihydroxyhept 6 enoic acid" OR (crestor) OR (rosuvas) OR (rosuvastatin calcium) OR (s 4522) OR (s4522) OR (zd 4522) OR (zd4522)  #35 'pitavastatin'/exp OR "7 [2 cyclopropyl 4 (4 fluorophenyl) 3 quinolyl] 3, 5 dihydroxy 6 heptenoic acid" OR (alipza) OR (itavastatin) OR (itavastatin calcium) OR (livalo) OR (livazo) OR "monocalcium bis [7 [2 cyclopropyl 4 (4 fluorophenyl) 3 quinolyl] 3, 5 dihydroxy 6 heptenoate]" OR (nisvastatin) OR (nk 104) OR (nk104) OR (nks 104) OR (nks104) OR (pitava) OR (pitavastatin calcium) OR (ribar) OR (vezepra)  #36 'tenivastatin'/exp OR "7 [8 (2, 2 dimethylbutyryloxy) 2, 6 dimethyl 1, 2, 6, 7, 8, 8a hexahydronaphthalen 1 yl] 3, 5 dihydroxyheptanoic acid" OR "7 [8 [(2, 2 dimethylbutanoyl) oxy] 2, 6 dimethyl 1, 2, 6, 7, 8, 8a hexahydronaphthalen 1 yl] 3, 5 dihydroxyheptanoic acid" OR (tenivastatin calcium)  #37 'bervastatin'/exp OR "7 [4 (4 fluorophenyl) spiro [2h 1 benzopyran 2, 1 cyclopentan] 3 yl] 3, 5 dihydroxy 6 heptenoic acid ethyl ester" OR "ethyl 7 [4 (4 fluorophenyl) spiro [2h 1 benzopyran 2, 1 cyclopentan] 3 yl] 3, 5 dihydroxy 6 heptenoate" OR "ethyl 7 [4 (4 fluorophenyl) spiro [chromene 2, 1 cyclopentan] 3 yl] 3, 5 dihydroxyhept 6 enoate" OR (ls 2904) OR (ls2904)  #38 'compactin'/exp OR "1, 2, 3, 7, 8, 8a hexahydro 7 methyl 8 [2 (tetrahydro 4 hydroxy 6 oxo 2h pyran 2 yl) ethyl] 1 naphthyl 2 methylbutyrate" OR "2 methylbutyric acid 1, 2, 3, 7, 8, 8a hexahydro 7 methyl 8 [2 (tetrahydro 4 hydroxy 6 oxo 2h pyran 2 yl) ethyl] 1 naphthyl ester" OR "compactin (penicillium)" OR (cs 500) OR (cs500) OR (mevastatin) OR (mevastin) OR (ml 236b) OR (ml236b)  #39 'crilvastatin'/exp OR "(3, 3, 5 trimethylcyclohexyl) 5 oxopyrrolidine 2 carboxylate" OR "5 oxoproline 3, 3, 5 trimethylcyclohexyl ester" OR "5 oxopyrrolidine 2 carboxylic acid (3, 3, 5 trimethylcyclohexyl) ester" OR (pmd 387) OR (pmd387) OR "pyroglutamic acid 3, 3, 5 trimethylcyclohexyl ester"  #40 'dalvastatin'/exp OR "6 [2 [2 (4 fluoro 3 methylphenyl) 4, 4, 6, 6 tetramethyl 1 cyclohexenyl] vinyl] 3, 4, 5, 6 tetrahydro 4 hydroxy 2h pyran 2 one" OR (rg 12561) OR (rg-12561)  #41 'glenvastatin'/exp OR "6 [2 [4 (4 fluorophenyl) 2 (1 methylethyl) 6 phenyl 3 pyridinyl] ethenyl] tetrahydro 4 hydroxy 2h pyran 2 one" OR "6 [2 [4 (4 fluorophenyl) 2 isopropyl 6 phenyl 3 pyridyl] vinyl] 3, 4, 5, 6 tetrahydro 4 hydroxy 2h pyran 2 one" OR (hr 780) OR (hr780)  #42 'mevinolinic acid'/exp OR "7 [1, 2, 6, 7, 8, 8a hexahydro 2, 6 dimethyl 8 (2 methylbutyryloxy) 1 naphthyl] 3, 5 dihydroxyheptanoic acid" OR (mevinolinate)  #43 'monacolin J'/exp  #44 'monacolin L'/exp  #45 'ezetimibe plus rosuvastatin'/exp OR (ezetimibe-rosuvastatin) OR (ezetimibe rosuvastatin) OR (rosuvastatin plus ezetimibe) OR (rosuvastatin-ezetimibe) OR (rosuvastatin ezetimibe)  #46 'ezetimibe plus simvastatin'/exp OR (ezetimibe simvastatin combination) OR (ezetimibe, simvastatin drug combination) OR (ezetimibe-simvastatin) OR (ezetimibe-simvastatin combination) OR (ezetimibe simvastatin) OR (inegy) OR (simvastatin plus ezetimibe) OR (simvastatin-ezetimibe) OR (simvastatin ezetimibe) OR (vytorin) OR (zetsim) OR (zintrepid)  #47 'fenofibrate plus pravastatin'/exp OR (fenofibrate plus pravastatin sodium) OR (pravafenix) OR (pravastatin plus fenofibrate) OR (pravastatin sodium plus fenofibrate)  #48 'fenofibrate plus simvastatin'/exp OR (cholib) OR (fenofibrate simvastatin) OR (simvastatin plus fenofibrate) OR (simvastatin fenofibrate)  #49 'fenofibric acid plus rosuvastatin'/exp OR (certriad) OR (fenofibric acid plus rosuvastatin calcium) OR (rosuvastatin calcium plus fenofibric acid) OR (rosuvastatin plus fenofibric acid)  #50 #1 OR #2 OR #3 OR #4 OR #5 OR #6 OR #7 OR #8 OR #9 OR #10 OR #11 OR #12 OR #13 OR #14 OR #15 OR #16 OR #17 OR #18 OR #19 OR #20 OR #21 OR #22 OR #23 OR #24 OR #25 OR #26 OR #27 OR #28 OR #29 OR #30 OR #31 OR #32 OR #33 OR #34 OR #35 OR #36 OR #37 OR #38 OR #39 OR #40 OR #41 OR #42 OR #43 OR #44 OR #45 OR #46 OR #47 OR #48 OR #49  #51 'diabetic retinopathy'/exp OR (diabetes mellitus retinopathy) OR (diabetes retinopathy) OR (diabetic retinitis) OR (retinopathia diabetica) OR (retinopathy, diabetes)  #52 #50 AND #51  #53 #52 AND limit EMBASE |
| CENTRAL (via ELSEVIER) | #1 MeSH descriptor: [Hypolipidemic Agents] explode all trees  #2 (Hypolipidemic Agents) or (Agents, Hypolipidemic) or (Antihyperlipidemics) or (Hypolipidemic Drugs) or (Drugs, Hypolipidemic) or (Antilipemic Drugs) or (Drugs, Antilipemic) or (Antilipemics) or (Antihyperlipemics) or (Antilipemic Agents) or (Agents, Antilipemic)  #3 MeSH descriptor: [Fibric Acids] explode all trees  #4 (Fibric Acids) or (Methyl-2-Phenoxypropanoic Acid Derivatives) or (Methyl 2 Phenoxypropanoic Acid Derivatives) or (Fibric Acid Derivative) or (Acid Derivatives, Fibric) or (2-Phenoxy Isobutyric Acids) or (2 Phenoxy Isobutyric Acids) or (Isobutyric Acids, 2-Phenoxy) or (2-Phenoxy-2-Methylpropionic Acid Derivatives) or (2 Phenoxy 2 Methylpropionic Acid Derivatives) or (Fibrates)  #5 MeSH descriptor: [Fenofibrate] explode all trees  #6 (Fenofibrate) or (Procetofen) or (Procetofene) or (Phenofibrate) or (Apo-Feno-Micro) or (Apo Feno Micro) or (Apotex Brand of Procetofen) or (Apo-Fenofibrate) or (Apo Fenofibrate) or (CiL) or (Lichtenstein Brand of Procetofen) or (Controlip) or (Knoll Brand of Procetofen) or (durafenat) or (Merck dura Brand of Procetofen) or (Fenobeta) or (betapharm Brand of Procetofen) or (Fenofanton) or (Q-Pharm Brand of Procetofen) or (Q Pharm Brand of Procetofen) or (Anto Brand of Procetofen) or (Fenofibrat AbZ) or (AbZ Brand of Procetofen) or (Fenofibrat AL) or (Aliud Brand of Procetofen) or (Fenofibrat AZU) or (AZU, Fenofibrat) or (Azupharma Brand of Procetofen) or (Fenofibrat Heumann) or (Heumann, Fenofibrat) or (Heumann Brand of Procetofen) or (Fenofibrat Hexal) or (Hexal, Fenofibrat) or (Hexal Brand of Procetofen) or (Fenofibrat Stada) or (Stada, Fenofibrat) or (Stadapharm Brand of Procetofen) or (fenofibrat von ct) or (ct-Arzneimittel Brand of Procetofen) or (ct Arzneimittel Brand of Procetofen) or (Fenofibrat-ratiopharm) or (Fenofibrat ratiopharm) or (ratiopharm Brand of Procetofen) or (Fénofibrate MSD) or (GNR-Pharma Brand of Procetofen) or (GNR Pharma Brand of Procetofen) or (Gen-Fenofibrate) or (Gen Fenofibrate) or (Genpharm Brand of Procetofen) or (LF-178) or (LF 178) or (LF178) or (Lipanthyl) or (Fenofibrat FPh) or (Fournier Brand of Procetofen) or (Fénofibrate Debat) or (Debat, Fénofibrate) or (Supralip) or (Lipidil) or (Lipidil-Ter) or (Lipidil Ter) or (Schering-Plough Brand of Procetofen) or (Schering Plough Brand of Procetofen) or (Secalip) or (United Drug Brand of Procetofen) or (Lipantil) or (Liparison) or (Novartis Brand of Procetofen) or (Livesan) or (Bouchara Brand of Procetofen) or (Lofibra) or (Gate Brand of Procetofen) or (MTW-Fenofibrat) or (MTW Fenofibrat) or (MTW Brand of Procetofen) or (Novo-Fenofibrate) or (Novo Fenofibrate) or (Novopharm Brand of Procetofen) or (Nu-Fenofibrate) or (Nu Fenofibrate) or (Nu-Pharm Brand of Procetofen) or (Nu Pharm Brand of Procetofen) or (PMS-Fenofibrate Micro) or (PMS Fenofibrate Micro) or (Pharmascience Brand of Procetofen) or (Tricor) or (Normalip) or (Abbott Brand of Procetofen) or (Antara Micronized Procetofen) or (Micronized Procetofen, Antara) or (Procetofen, Antara Micronized) or (Reliant Brand of Procetofen) or (Procetofen Reliant Brand) or (Lipanon)  #7 MeSH descriptor: [Bezafibrate] explode all trees  #8 (Bezafibrate) or (Befibrat) or (Hennig Brand of Bezafibrate) or (Bezafibrate Hennig Brand) or (Beza-Lande) or (Beza Lande) or (BezaLande) or (Synthelabo Brand of Bezafibrate) or (Bezafibrate Synthelabo Brand) or (Beza-Puren) or (Beza Puren) or (BezaPuren) or (Isis Brand of Bezafibrate) or (Bezafibrate Isis Brand) or (Bezabeta) or (Betapharm Brand of Bezafibrate) or (Bezafibrate Betapharm Brand) or (Bezacur) or (Hexal Brand of Bezafibrate) or (Bezafibrate Hexal Brand) or (Bezafibrat PB) or (PB, Bezafibrat) or (Teva Brand of Bezafibrate) or (Bezafibrate Teva Brand) or (Bezafisal) or (Cryopharma Brand of Bezafibrate) or (Bezafibrate Cryopharma Brand) or (Bezalip) or (Eulitop) or (Lakeside Brand of Bezafibrate) or (Bezafibrate Lakeside Brand) or (Boehringer Mannheim Brand of Bezafibrate) or (Bezamerck) or (durabezur) or (BM-15.075) or (BM 15.075) or (BM15.075) or (Cedur) or (Difaterol) or (Roche Brand of Bezafibrate) or (Bezafibrate Roche Brand) or (Béfizal) or (Lipox) or (TAD Brand of Bezafibrate) or (Bezafibrate TAD Brand) or (Reducterol) or (Elfar Brand of Bezafibrate) or (Bezafibrate Elfar Brand) or (Regadrin B) or (Berlin-Chemie Brand of Bezafibrate) or (Berlin Chemie Brand of Bezafibrate) or (Bezafibrate Berlin-Chemie Brand) or (Sklerofibrat) or (Merckle Brand of Bezafibrate) or (Bezafibrate Merckle Brand) or (Solibay) or (Bayer Brand of Bezafibrate) or (Bezafibrate Bayer Brand) or (Azufibrat) or (Azupharma Brand of Bezafibrate) or (Bezafibrate Azupharma Brand)  #9 MeSH descriptor: [Clofibrate] explode all trees  #10 (Clofibrate) or (Ethyl Chlorophenoxyisobutyrate) or (Chlorophenoxyisobutyrate, Ethyl) or (Clofibric Acid, Ethyl Ester) or (Atromid) or (Atromid S) or (Miscleron) or (Miskleron) or (Athromidin) OR (ciprofibrate) or (Oroxadin) or (Win 35,833) or (Lipanor) or (Modalim) or (Hyperlipen) OR(Clofenapate) or (Methylclofenapate) or (Methyl Clofenapate) or (Clofenapate, Methyl) or "ICI-55695" or "ICI 55695" or "ICI55695"  #11 MeSH descriptor: [Clofenapate] explode all trees  #12 MeSH descriptor: [Gemfibrozil] explode all trees  #13 (Gemfibrozil) or (Gemfibrosil) or (Ausgem) or (Sigma Brand of Gemfibrozil) or (Bolutol) or (Farmasierra Brand of Gemfibrozil) or (Chem mart Gemfibrozil) or (Chem mart Brand of Gemfibrozil) or (CI-719) or (CI 719) or (CI719) or (DBL Gemfibrozil) or (Bull Brand of Gemfibrozil) or (Decrelip) or (Ferrer Brand of Gemfibrozil) or (Gemfi 1A Pharma) or (1A Brand of Gemfibrozil) or (Gemfibrozilo Bayvit) or (Bayvit, Gemfibrozilo) or (Bayvit Brand of Gemfibrozil) or (Gemfibrozilo Bexal) or (Bexal Brand of Gemfibrozil) or (Gemfibrozilo Ur) or (Cantabria Brand of Gemfibrozil) or (Gemhexal) or (Hexal Brand of Gemfibrozil) or (Gen-Gemfibrozil) or (Gen Gemfibrozil) or (GenGemfibrozil) or (Genpharm Brand of Gemfibrozil) or (GenRX Gemfibrozil) or (Gemfibrozil, GenRX) or (Faulding Brand of Gemfibrozil) or (Healthsense Gemfibrozil) or (Gemfibrozil, Healthsense) or (Healthsense Brand of Gemfibrozil) or (Jezil) or (Alphapharm Brand of Gemfibrozil) or (Lipazil) or (Douglas Brand of Gemfibrozil) or (Lipox Gemf) or (TAD Brand of Gemfibrozil) or (Litarek) or (Ipsen Brand of Gemfibrozil) or (Lopid) or (Lopid R) or (Warner-Lambert Brand of Gemfibrozil) or (Warner Lambert Brand of Gemfibrozil) or (Pfizer Brand of Gemfibrozil) or (United Drug Brand of Gemfibrozil) or (Lipur) or (Parke Davis Brand of Gemfibrozil) or (Novo-Gemfibrozil) or (Novo Gemfibrozil) or (Novopharm Brand of Gemfibrozil) or (Nu-Gemfibrozil) or (Nu Gemfibrozil) or (NuGemfibrozil) or (Nu-Pharm Brand of Gemfibrozil) or (Nu Pharm Brand of Gemfibrozil) or (Pilder) or (Quimifar Brand of Gemfibrozil) or (PMS-Gemfibrozil) or (PMS Gemfibrozil) or (Pharmascience Brand of Gemfibrozil) or (SBPA Gemfibrozil) or (Gemfibrozil, SBPA) or (Biochemie Brand of Gemfibrozil) or (Terry White Chemists Gemfibrozil) or (Terry White Chemists Brand of Gemfibrozil) or (Trialmin) or (Menarini Brand of Gemfibrozil) or (Apo-Gemfibrozil) or (Apo Gemfibrozil) or (ApoGemfibrozil) or (Apotex Brand of Gemfibrozil)  #14 (simfibrate) or "1,3-propanediol bis(alpha-p-chlorophenoxyisobutyrate)" or (CLY 503) or (CLY-503) or (Cholesolvin) OR (etofibrate) or (Lipo-Merz) or (Tricerol) or (etofibrate hydrochloride) OR (clofibride) or "N-dimethylbutyramide 4-chlorophenoxyisobutyrate" or "4-hydroxy-N,N-dimethylbutyramide 4-chlorophenoxyisobutyrate" or "MG 46" or (lipenan) OR (pirifibrate) or (Bratenol) or "EL-466" or "(6-(hydroxymethyl)-2-pyridyl)methyl-2-(4-chlorophenoxy)-2-methyl-propionate hydrochloride" OR (clinofibrate) or "1,1-bis(4'-(1''-carboxy-1''-methylpropoxy)phenyl)cyclohexane" or "S 8527" or "clinofibrate, (R*,R*)-(-)-isomer" or "clinofibrate, (R*,S*)-isomer" or "clinofibrate, (R*,R*)-(+)-isomer" or "clinofibrate, (R*,R*)-(+-)-isomer" OR (dulofibrate) or "4-chlorophenyl 2-(4-chlorophenoxy)isobutyrate" or "2-(4-chlorophenoxy)isobutyric acid 4-chlorophenyl ester" OR (lifibrate) or "1-methyl-4-piperidyl bis(4-chlorophenoxy)acetate" or "SaH 42-348" OR (picafibrate) or "(4-chloro-2-phenoxy)-2-methyl-2-propionyl-2- ethylnicotinamide" or "F 1393" OR ronifibrate or biclofibrate or fenirofibrate or nicofibrate or ponfibrate or salafibrate or serfibrate or tiafibrate or timofibrate OR (tocofibrate) or "tocopheryl 2-(p-chlorophenoxy)isobutyrate"  #15 MeSH descriptor: [Hydroxymethylglutaryl-CoA Reductase Inhibitors] explode all trees  #16 (Hydroxymethylglutaryl-CoA Reductase Inhibitors) or (Hydroxymethylglutaryl CoA Reductase Inhibitors) or (Inhibitors, Hydroxymethylglutaryl-CoA Reductase) or (Reductase Inhibitors, Hydroxymethylglutaryl-CoA) or (Inhibitors, HMG-CoA Reductase) or (Inhibitors, HMG CoA Reductase) or (Inhibitors, Hydroxymethylglutaryl-Coenzyme A) or (Hydroxymethylglutaryl-Coenzyme A Inhibitors) or (Inhibitors, Hydroxymethylglutaryl Coenzyme A) or (Statins, HMG-CoA) or (HMG-CoA Statins) or (Statins, HMG CoA) or (HMG-CoA Reductase Inhibitors) or (HMG CoA Reductase Inhibitors) or (Reductase Inhibitors, HMG-CoA) or (Inhibitors, Hydroxymethylglutaryl-CoA) or (Hydroxymethylglutaryl-CoA Inhibitors) or (Inhibitors, Hydroxymethylglutaryl CoA)  #17 MeSH descriptor: [Simvastatin] explode all trees  #18 (Simvastatin) or (Synvinolin) or (MK-733) or (MK 733) or (MK733) or (Zocor) or (SINVALIP) or (VASLIP) OR (ezetimibe, simvastatin drug combination) or (ezetimibe-simvastatin combination) or (Vytorin) or (Inegy) OR (Lovastatin) or (Mevinolin) or (Monacolin K) or (6-Methylcompactin) or (6 Methylcompactin) or (MK-803) or (MK 803) or (MK803) or (Mevacor) or "Lovastatin, (1 alpha(S*))-Isomer" or (Lovastatin, 1 alpha-Isomer) or (1 alpha-Isomer Lovastatin) or (Lovastatin, 1 alpha Isomer) or (alpha-Isomer Lovastatin, 1)  #19 MeSH descriptor: [Ezetimibe, Simvastatin Drug Combination] explode all trees  #20 MeSH descriptor: [Lovastatin] explode all trees  #21 MeSH descriptor: [Pravastatin] explode all trees  #22 (Pravastatin) or (Eptastatin) or (Vasten) or (Aventis Brand of Pravastatin Sodium) or (CS-514) or (CS 514) or (CS514) or (Lin-Pravastatin) or (Lin Pravastatin) or (Linson Pharma Brand of Pravastatin Sodium) or (Lipemo) or (Squibb Brand of Pravastatin Sodium) or (Liplat) or (Esteve Brand of Pravastatin Sodium) or (Nu-Pravastatin) or (Nu Pravastatin) or (Nu-Pharma Brand of Pravastatin Sodium) or (Prareduct) or (Sankyo Brand of Pravastatin Sodium) or (Mevalotin) or (Pravachol) or (Elisor) or (Selektine) or (Pravacol) or (Pravasin) or (Bristol-Myers Squibb Brand of Pravastatin Sodium) or (Lipostat) or (Pravastatin Monosodium Salt, (6 beta) -Isomer) or (Pravastatin Sodium) or (Pravastatin Sodium Salt) or (Sodium Salt, Pravastatin) or (Pravastatin tert-Octylamine Salt) or (Pravastatin tert Octylamine Salt) or (Pravastatin, (6 beta) -Isomer) or (RMS-431) or (RMS 431) or (RMS431) or (SQ-31000) or (SQ 31000) or (SQ31000) or (SQ-31,000) or (SQ 31,000) or (SQ31,000) or (Apo-Pravastatin) or (Apo Pravastatin) or (Apotex Brand of Pravastatin Sodium) or (Bristacol) or (Juste Brand of Pravastatin Sodium)  #23 (fluvastatin) or (fluindostatin) or "7-(3-(4-fluorophenyl)-1-(1-methylethyl)-1H-indol-2-yl)-3,5-dihydroxy-6-heptenoate" or (Lescol) or (XU 62-320) or (XU-62320) or (XU 62320) or (fluvastatin sodium) or (fluvastatin sodium salt)  #24 MeSH descriptor: [Atorvastatin Calcium] explode all trees  #25 (atorvastatin) or (liptonorm) or (Lipitor) or (CI 981) or (CI-981) or (atorvastatin calcium) or (atorvastatin calcium hydrate) or (atorvastatin calcium trihydrate) or (atorvastatin, calcium salt) or (atorvastatin calcium anhydrous)  #26 (cerivastatin) or (Kazak) or (cerivastatin sodium) or "6-Heptenoic acid, 7-(4-(4-fluorophenyl)-5-(methoxymethyl)-2,6-bis(1-methylethyl)-3-pyridinyl)-3,5-dihydroxy-, monosodium salt, (S-(R*,S*-(E)))-" or "7-(4-(4-fluorophenyl)-2,6-diisopropyl-5-(methoxymethyl)pyrid-3-yl)-3,5-dihydroxy-6-heptenoate sodium salt" or (rivastatin) or (Certa) or (Bay w 6228) or (Baycol) or (Lipobay) OR rosuvastatin  #27 MeSH descriptor: [Rosuvastatin Calcium] explode all trees  #28 (rosuvastatin) or (rosuvastatin calcium) or (ZD4522) or (ZD 4522) or (Crestor) OR (pitavastatin) or (nisvastatin) or (itavastatin) or (P 872441) or (P-872441) or (NK 104) or (NK-104) OR (mevastatin) or (compactin) or "6-demethylmevinolin" or "CS 500" or "ML 236B" or "ML236B" or "ML-236B" OR crilvastatin OR (dalvastatin) or "6-(2-(2-(4-fluoro-3-methylphenyl)-4,4,6,6-tetramethyl-1-cyclohexen-1-yl)ethenyl)tetrahydro-4-hydroxy-2H-pyran-2-one" or "RG 12561" or "RG-12561" OR glenvastatin  #29 (mevinolinic acid) or (lovestatin acid) or "mevinolinic acid, (1S-(1alpha(betaS*,deltaS*),2alpha,6beta,8beta(R*),8aalpha))-isomer" or "mevinolinic acid, aluminum salt (3:1)" or "mevinolinic acid, monoammonium salt, 1S-(1alpha(betaS*),2alpha,6beta,8beta(R*),8aalpha)-isomer" or (mevinolinic acid, monosodium salt) or "mevinolinic acid, monosodium salt, (1S-(1alpha(betaS*,deltaS*),2alpha,6beta,8beta(R*),8aalpha))-isomer" or "L 154819" or "L-154819" or "L-154,819" or "mevinolinic acid, calcium salt (2:1)" OR (monacolin J) OR (monacolin L)  #30 #1 or #2 or #3 or #4 or #5 or #6 or #7 or #8 or #9 or #10 or #11 or #12 or #13 or #14 or #15 or #16 or #17 or #18 or #19 or #20 or #21 or #22 or #23 or #24 or #25 or #26 or #27 or #28 or #29  #31 MeSH descriptor: [Diabetic Retinopathy] explode all trees  #32 (Diabetic Retinopathy) or (Diabetic Retinopathies) or (Retinopathies, Diabetic) or (Retinopathy, Diabetic)  #33 #31 or #32  #34 #30 and #33 |
| LILACS (via BVS - Biblioteca Virtual da Saúde) | MH:Hipolipemiantes OR Hipolipemiantes OR (Hypolipidemic Agents) OR MH:D27.505.519.186.071$ OR MH:D27.505.954.557.500$ OR (Agents, Hypolipidemic) OR (Antihyperlipidemics) OR (Hypolipidemic Drugs) OR (Drugs, Hypolipidemic) OR (Antilipemic Drugs) OR (Drugs, Antilipemic) OR (Antilipemics) OR (Antihyperlipemics) OR (Antilipemic Agents) OR (Agents, Antilipemic) OR  MH:"Acidos FÌbricos" OR (¡cidos FÌbricos) OR (Fibric Acids) OR MH:D02.241.081.114.968.500$ OR MH:D02.355.726.305$ OR MH:D02.455.426.559.389.657.654.305$ OR (Methyl-2-Phenoxypropanoic Acid Derivatives) OR (Methyl 2 Phenoxypropanoic Acid Derivatives) OR (Fibric Acid Derivative) OR (Acid Derivatives, Fibric) OR (2-Phenoxy Isobutyric Acids) OR (2 Phenoxy Isobutyric Acids) OR (Isobutyric Acids, 2-Phenoxy) OR (2-Phenoxy-2-Methylpropionic Acid Derivatives) OR (2 Phenoxy 2 Methylpropionic Acid Derivatives) OR (Fibrates) OR MH:Fenofibrato OR Fenofibrat$ OR MH:D02.241.081.114.968.500.625$ OR MH:D02.355.726.305.625$ OR MH:D02.455.426.559.389.115.124.750$ OR MH:D02.455.426.559.389.657.654.305.625$ OR MH:D02.522.223.750$ OR (Procetofen) OR (Procetofene) OR (Phenofibrate) OR (Apo-Feno-Micro) OR (Apo Feno Micro) OR (Apotex Brand of Procetofen) OR (Apo-Fenofibrate) OR (Apo Fenofibrate) OR (CiL) OR (Lichtenstein Brand of Procetofen) OR (Controlip) OR (Knoll Brand of Procetofen) OR (durafenat) OR (Merck dura Brand of Procetofen) OR (Fenobeta) OR (betapharm Brand of Procetofen) OR (Fenofanton) OR (Q-Pharm Brand of Procetofen) OR (Q Pharm Brand of Procetofen) OR (Anto Brand of Procetofen) OR (Fenofibrat AbZ) OR (AbZ Brand of Procetofen) OR (Fenofibrat AL) OR (Aliud Brand of Procetofen) OR (Fenofibrat AZU) OR (AZU, Fenofibrat) OR (Azupharma Brand of Procetofen) OR (Fenofibrat Heumann) OR (Heumann, Fenofibrat) OR (Heumann Brand of Procetofen) OR (Fenofibrat Hexal) OR (Hexal, Fenofibrat) OR (Hexal Brand of Procetofen) OR (Fenofibrat Stada) OR (Stada, Fenofibrat) OR (Stadapharm Brand of Procetofen) OR (fenofibrat von ct) OR (ct-Arzneimittel Brand of Procetofen) OR (ct Arzneimittel Brand of Procetofen) OR (Fenofibrat-ratiopharm) OR (Fenofibrat ratiopharm) OR (ratiopharm Brand of Procetofen) OR (FÈnofibrate MSD) OR (GNR-Pharma Brand of Procetofen) OR (GNR Pharma Brand of Procetofen) OR (Gen-Fenofibrate) OR (Gen Fenofibrate) OR (Genpharm Brand of Procetofen) OR (LF-178) OR (LF 178) OR (LF178) OR (Lipanthyl) OR (Fenofibrat FPh) OR (Fournier Brand of Procetofen) OR (FÈnofibrate Debat) OR (Debat, FÈnofibrate) OR (Supralip) OR (Lipidil) OR (Lipidil-Ter) OR (Lipidil Ter) OR (Schering-Plough Brand of Procetofen) OR (Schering Plough Brand of Procetofen) OR (Secalip) OR (United Drug Brand of Procetofen) OR (Lipantil) OR (Liparison) OR (Novartis Brand of Procetofen) OR (Livesan) OR (Bouchara Brand of Procetofen) OR (Lofibra) OR (Gate Brand of Procetofen) OR (MTW-Fenofibrat) OR (MTW Fenofibrat) OR (MTW Brand of Procetofen) OR (Novo-Fenofibrate) OR (Novo Fenofibrate) OR (Novopharm Brand of Procetofen) OR (Nu-Fenofibrate) OR (Nu Fenofibrate) OR (Nu-Pharm Brand of Procetofen) OR (Nu Pharm Brand of Procetofen) OR (PMS-Fenofibrate Micro) OR (PMS Fenofibrate Micro) OR (Pharmascience Brand of Procetofen) OR (Tricor) OR (Normalip) OR (Abbott Brand of Procetofen) OR (Antara Micronized Procetofen) OR (Micronized Procetofen, Antara) OR (Procetofen, Antara Micronized) OR (Reliant Brand of Procetofen) OR (Procetofen Reliant Brand) OR (Lipanon) OR  MH:Bezafibrato OR Bezafibrat$ OR MH:D02.065.277.067$ OR MH:D02.241.081.114.968.500.249$ OR MH:D02.241.223.100.100.120$ OR MH:D02.241.223.100.200.249$ OR MH:D02.355.726.305.249$ OR MH:D02.455.426.559.389.127.085.101$ OR MH:D02.455.426.559.389.127.250.249$ OR MH:D02.455.426.559.389.657.654.305.249$ OR (Befibrat) OR (Hennig Brand of Bezafibrate) OR (Bezafibrate Hennig Brand) OR (Beza-Lande) OR (Beza Lande) OR (BezaLande) OR (Synthelabo Brand of Bezafibrate) OR (Bezafibrate Synthelabo Brand) OR (Beza-Puren) OR (Beza Puren) OR (BezaPuren) OR (Isis Brand of Bezafibrate) OR (Bezafibrate Isis Brand) OR (Bezabeta) OR (Betapharm Brand of Bezafibrate) OR (Bezafibrate Betapharm Brand) OR (Bezacur) OR (Hexal Brand of Bezafibrate) OR (Bezafibrate Hexal Brand) OR (Bezafibrat PB) OR (PB, Bezafibrat) OR (Teva Brand of Bezafibrate) OR (Bezafibrate Teva Brand) OR (Bezafisal) OR (Cryopharma Brand of Bezafibrate) OR (Bezafibrate Cryopharma Brand) OR (Bezalip) OR (Eulitop) OR (Lakeside Brand of Bezafibrate) OR (Bezafibrate Lakeside Brand) OR (Boehringer Mannheim Brand of Bezafibrate) OR (Bezamerck) OR (durabezur) OR (BM-15.075) OR (BM 15.075) OR (BM15.075) OR (Cedur) OR (Difaterol) OR (Roche Brand of Bezafibrate) OR (Bezafibrate Roche Brand) OR (BÈfizal) OR (Lipox) OR (TAD Brand of Bezafibrate) OR (Bezafibrate TAD Brand) OR (Reducterol) OR (Elfar Brand of Bezafibrate) OR (Bezafibrate Elfar Brand) OR (Regadrin B) OR (Berlin-Chemie Brand of Bezafibrate) OR (Berlin Chemie Brand of Bezafibrate) OR (Bezafibrate Berlin-Chemie Brand) OR (Sklerofibrat) OR (Merckle Brand of Bezafibrate) OR (Bezafibrate Merckle Brand) OR (Solibay) OR (Bayer Brand of Bezafibrate) OR (Bezafibrate Bayer Brand) OR (Azufibrat) OR (Azupharma Brand of Bezafibrate) OR (Bezafibrate Azupharma Brand) OR  MH:Clofibrato OR Clofibrat$ OR MH:D02.241.081.114.968.500.500.195$ OR MH:D02.355.726.305.500.195$ OR MH:D02.455.426.559.389.657.654.305.500.195$ OR (Ethyl Chlorophenoxyisobutyrate) OR (Chlorophenoxyisobutyrate, Ethyl) OR (Clofibric Acid, Ethyl Ester) OR (Atromid) OR (Atromid S) OR (Miscleron) OR (Miskleron) OR (Athromidin) OR  ciprofibrat$ OR (Oroxadin) OR (Win 35,833) OR (Lipanor) OR (Modalim) OR (Hyperlipen) OR MH:Clofenapato OR Clofenapat$ OR MH:D02.241.081.114.968.500.374$ OR MH:D02.355.726.305.374$ OR MH:D02.455.426.559.389.185.210$ OR MH:D02.455.426.559.389.657.654.305.374$ OR (Methylclofenapate) OR (Methyl Clofenapate) OR (Clofenapate, Methyl) OR "ICI-55695" OR "ICI 55695" OR "ICI55695" OR MH:Genfibrozila OR Genfibrozi$ OR MH:D02.241.081.114.968.500.750$ OR MH:D02.241.081.944.509.350$ OR MH:D02.355.726.305.750$ OR MH:D02.455.426.559.389.657.654.305.750$ OR MH:D10.251.400.895.593.350$ OR (Gemfibrosil) OR (Ausgem) OR (Sigma Brand of Gemfibrozil) OR (Bolutol) OR (Farmasierra Brand of Gemfibrozil) OR (Chem mart Gemfibrozil) OR (Chem mart Brand of Gemfibrozil) OR (CI-719) OR (CI 719) OR (CI719) OR (DBL Gemfibrozil) OR (Bull Brand of Gemfibrozil) OR (Decrelip) OR (Ferrer Brand of Gemfibrozil) OR (Gemfi 1A Pharma) OR (1A Brand of Gemfibrozil) OR (Gemfibrozilo Bayvit) OR (Bayvit, Gemfibrozilo) OR (Bayvit Brand of Gemfibrozil) OR (Gemfibrozilo Bexal) OR (Bexal Brand of Gemfibrozil) OR (Gemfibrozilo Ur) OR (Cantabria Brand of Gemfibrozil) OR (Gemhexal) OR (Hexal Brand of Gemfibrozil) OR (Gen-Gemfibrozil) OR (Gen Gemfibrozil) OR (GenGemfibrozil) OR (Genpharm Brand of Gemfibrozil) OR (GenRX Gemfibrozil) OR (Gemfibrozil, GenRX) OR (Faulding Brand of Gemfibrozil) OR (Healthsense Gemfibrozil) OR (Gemfibrozil, Healthsense) OR (Healthsense Brand of Gemfibrozil) OR (Jezil) OR (Alphapharm Brand of Gemfibrozil) OR (Lipazil) OR (Douglas Brand of Gemfibrozil) OR (Lipox Gemf) OR (TAD Brand of Gemfibrozil) OR (Litarek) OR (Ipsen Brand of Gemfibrozil) OR (Lopid) OR (Lopid R) OR (Warner-Lambert Brand of Gemfibrozil) OR (Warner Lambert Brand of Gemfibrozil) OR (Pfizer Brand of Gemfibrozil) OR (United Drug Brand of Gemfibrozil) OR (Lipur) OR (Parke Davis Brand of Gemfibrozil) OR (Novo-Gemfibrozil) OR (Novo Gemfibrozil) OR (Novopharm Brand of Gemfibrozil) OR (Nu-Gemfibrozil) OR (Nu Gemfibrozil) OR (NuGemfibrozil) OR (Nu-Pharm Brand of Gemfibrozil) OR (Nu Pharm Brand of Gemfibrozil) OR (Pilder) OR (Quimifar Brand of Gemfibrozil) OR (PMS-Gemfibrozil) OR (PMS Gemfibrozil) OR (Pharmascience Brand of Gemfibrozil) OR (SBPA Gemfibrozil) OR (Gemfibrozil, SBPA) OR (Biochemie Brand of Gemfibrozil) OR (Terry White Chemists Gemfibrozil) OR (Terry White Chemists Brand of Gemfibrozil) OR (Trialmin) OR (Menarini Brand of Gemfibrozil) OR (Apo-Gemfibrozil) OR (Apo Gemfibrozil) OR (ApoGemfibrozil) OR (Apotex Brand of Gemfibrozil) OR  sinfibrato OR (simfibrate) OR "1,3-propanediol bis(alpha-p-chlorophenoxyisobutyrate)" OR (CLY 503) OR (CLY-503) OR (Cholesolvin) OR etofibrat$ OR (Lipo-Merz) OR (Tricerol) OR (etofibrate hydrochloride) OR  clofibrid$ OR "N-dimethylbutyramide 4-chlorophenoxyisobutyrate" OR "4-hydroxy-N,N-dimethylbutyramide 4-chlorophenoxyisobutyrate" OR "MG 46" OR (lipenan) OR  pirifibrat$ OR (Bratenol) OR "EL-466" OR "(6-(hydroxymethyl)-2-pyridyl)methyl-2-(4-chlorophenoxy)-2-methyl-propionate hydrochloride" OR dulofibrat$ OR "4-chlorophenyl 2-(4-chlorophenoxy)isobutyrate" OR "2-(4-chlorophenoxy)isobutyric acid 4-chlorophenyl ester" OR  clinofibrat$ OR "1,1-bis(4'-(1''-carboxy-1''-methylpropoxy)phenyl)cyclohexane" OR "S 8527" OR "clinofibrate, (R*,R*)-(-)-isomer" OR "clinofibrate, (R*,S*)-isomer" OR "clinofibrate, (R*,R*)-(+)-isomer" OR "clinofibrate, (R*,R*)-(+-)-isomer" OR  lifibrat$ OR "1-methyl-4-piperidyl bis(4-chlorophenoxy)acetate" OR "SaH 42-348" OR picafibrat$ OR "(4-chloro-2-phenoxy)-2-methyl-2-propionyl-2- ethylnicotinamide" OR "F 1393" OR  ronifibrat$ OR biclofibrat$ OR fenirofibrat$ OR nicofibrat$ OR ponfibrat$ OR salafibrat$ OR serfibrat$ OR tiafibrat$ OR timofibrat$ OR tocofibrat$ OR "tocopheryl 2-(p-chlorophenoxy)isobutyrate" OR  MH:"Inibidores de Hidroximetilglutaril-CoA Redutases" OR (Inibidores de Hidroximetilglutaril-CoA Redutases) OR (Hydroxymethylglutaryl-CoA Reductase Inhibitors) OR (Inhibidores de Hidroximetilglutaril-CoA Reductasas) OR MH:D27.505.519.186.071.202.370$ OR MH:D27.505.519.389.370$ OR MH:D27.505.954.557.500.202.370$ OR (Inhibitors, Hydroxymethylglutaryl-CoA Reductase) OR (Reductase Inhibitors, Hydroxymethylglutaryl-CoA) OR (Inhibitors, HMG-CoA Reductase) OR (Inhibitors, HMG CoA Reductase) OR (Inhibitors, Hydroxymethylglutaryl-Coenzyme A) OR (Hydroxymethylglutaryl-Coenzyme A Inhibitors) OR (Inhibitors, Hydroxymethylglutaryl Coenzyme A) OR (Statins, HMG-CoA) OR (HMG-CoA Statins) OR (Statins, HMG CoA) OR (HMG-CoA Reductase Inhibitors) OR (HMG CoA Reductase Inhibitors) OR (Reductase Inhibitors, HMG-CoA) OR (Inhibitors, Hydroxymethylglutaryl-CoA) OR(Hydroxymethylglutaryl-CoA Inhibitors) OR (Inhibitors, Hydroxymethylglutaryl CoA) OR  MH:Sinvastatina OR Sinvastati$ OR Simvastati$ OR MH:D02.455.426.559.847.638.400.900$ OR MH:D04.615.638.400.900$ OR (Synvinolin) OR (MK-733) OR (MK 733) OR (MK733) OR (Zocor) OR (SINVALIP) OR (VASLIP) OR (ezetimiba, sinvastatina combinaÁ„o) OR (ezetimiba-sinvastatina combinaÁ„o) OR (ezetimibe, simvastatin drug combination) OR (ezetimibe-simvastatin combination) OR (Vytorin) OR (Inegy) OR  MH:Lovastatina OR Lovastati$ OR MH:D02.455.426.559.847.638.400$ OR MH:D04.615.638.400$ OR (Mevinolin) OR (Monacolin K) OR (6-Methylcompactin) OR (6 Methylcompactin) OR (MK-803) OR (MK 803) OR (MK803) OR (Mevacor) OR "Lovastatin, (1 alpha(S*))-Isomer" OR (Lovastatin, 1 alpha-Isomer) OR (1 alpha-Isomer Lovastatin) OR (Lovastatin, 1 alpha Isomer) OR (alpha-Isomer Lovastatin, 1) OR  MH:Pravastatina OR Pravastati$ OR MH:D02.455.426.559.847.638.930$ OR MH:D04.615.638.930$ OR (Eptastatin) OR (Vasten) OR (Aventis Brand of Pravastatin Sodium) OR (CS-514) OR (CS 514) OR (CS514) OR (Lin-Pravastatin) OR (Lin Pravastatin) OR (Linson Pharma Brand of Pravastatin Sodium) OR (Lipemo) OR (Squibb Brand of Pravastatin Sodium) OR (Liplat) OR (Esteve Brand of Pravastatin Sodium) OR (Nu-Pravastatin) OR (Nu Pravastatin) OR (Nu-Pharma Brand of Pravastatin Sodium) OR (Prareduct) OR (Sankyo Brand of Pravastatin Sodium) OR (Mevalotin) OR (Pravachol) OR (Elisor) OR (Selektine) OR (Pravacol) OR (Pravasin) OR (Bristol-Myers Squibb Brand of Pravastatin Sodium) OR (Lipostat) OR (Pravastatin Monosodium Salt, (6 beta)-Isomer) OR (Pravastatin Sodium) OR (Pravastatin Sodium Salt) OR (Sodium Salt, Pravastatin) OR (Pravastatin tert-Octylamine Salt) OR (Pravastatin tert Octylamine Salt) OR (Pravastatin, (6 beta)-Isomer) OR (RMS-431) OR (RMS 431) OR (RMS431) OR (SQ-31000) OR (SQ 31000) OR (SQ31000) OR (SQ-31,000) OR (SQ 31,000) OR (SQ31,000) OR (Apo-Pravastatin) OR (Apo Pravastatin) OR (Apotex Brand of Pravastatin Sodium) OR (Bristacol) OR (Juste Brand of Pravastatin Sodium) OR fluvastatin$ OR (fluindostatin) OR "7-(3-(4-fluorophenyl)-1-(1-methylethyl)-1H-indol-2-yl)-3,5-dihydroxy-6-heptenoate" OR (Lescol) OR (XU 62-320) OR (XU-62320) OR (XU 62320) OR (fluvastatin sodium) OR (fluvastatin sodium salt) OR atorvastatin$ OR (liptonorm) OR (Lipitor) OR (CI 981) OR (CI-981) OR (atorvastatin calcium) OR (atorvastatin calcium hydrate) OR (atorvastatin calcium trihydrate) OR (atorvastatin, calcium salt) OR (atorvastatin calcium anhydrous) OR  cerivastatin$ OR (Kazak) OR (cerivastatin sodium) OR "6-Heptenoic acid, 7-(4-(4-fluorophenyl)-5-(methoxymethyl)-2,6-bis(1-methylethyl)-3-pyridinyl)-3,5-dihydroxy-, monosodium salt, (S-(R*,S*-(E)))-" OR "7-(4-(4-fluorophenyl)-2,6-diisopropyl-5-(methoxymethyl)pyrid-3-yl)-3,5-dihydroxy-6-heptenoate sodium salt" OR (rivastatin) OR (Certa) OR (Bay w 6228) OR (Baycol) OR (Lipobay) OR rosuvastatin$ OR (rosuvastatin calcium) OR (ZD4522) OR (ZD 4522) OR (Crestor) OR pitavastatin$ OR (nisvastatin) OR (itavastatin) OR (P 872441) OR (P-872441) OR (NK 104) OR (NK-104) OR mevastatin$ OR (compactin) OR "6-demethylmevinolin" OR "CS 500" OR "ML 236B" OR "ML236B" OR "ML-236B" OR crilvastatin$ OR dalvastatin$ OR "6-(2-(2-(4-fluoro-3-methylphenyl)-4,4,6,6-tetramethyl-1-cyclohexen-1-yl)ethenyl)tetrahydro-4-hydroxy-2H-pyran-2-one" OR "RG 12561" OR "RG-12561" OR (mevinolinic acid) OR (lovestatin acid) OR "mevinolinic acid, (1S-(1alpha(betaS*,deltaS*),2alpha,6beta,8beta(R*),8aalpha))-isomer" OR "mevinolinic acid, aluminum salt (3:1)" OR "mevinolinic acid, monoammonium salt, 1S-(1alpha(betaS*),2alpha,6beta,8beta(R*),8aalpha)-isomer" OR (mevinolinic acid, monosodium salt) OR "mevinolinic acid, monosodium salt, (1S-(1alpha(betaS*,deltaS*),2alpha,6beta,8beta(R*),8aalpha))-isomer" OR "L 154819" OR "L-154819" OR "L-154,819" OR "mevinolinic acid, calcium salt (2:1)" OR (monacolin J) OR (monacolin L) OR glenvastatin$  AND  MH:"Retinopatia Diabética" OR (Retinopatia Diabética) OR (Diabetic Retinopathy) OR (Retinopatía Diabética) OR MH:C11.768.257$ OR MH:C14.907.320.382$ OR MH:C19.246.099.500.382$ OR (Diabetic Retinopathies) OR (Retinopathies, Diabetic) OR (Retinopathy, Diabetic) |
